# Valorization of Wheat Milling Co-Products in *Carasau* Bread: Effects of Their Addition as Powders or After Fermentation on Physicochemical Properties, Volatile Profile and Sensory Characteristics

**DOI:** 10.3390/foods15203754

**Published:** 2026-10-21

**Authors:** Manuela Sanna, Valentina Tolu, Simonetta Fois, Marco Serra, Piero Pasqualino Piu, Daniela Piras, Vanna Sanna, Antonio Loddo, Tonina Roggio, Pasquale Catzeddu

**Affiliations:** 1 Porto Conte Ricerche, Loc. Tramariglio, 07041 Alghero, SS, Italy; tolu@portocontericerche.it (V.T.); fois@portocontericerche.it (S.F.); serram@portocontericerche.it (M.S.); sannav@portocontericerche.it (V.S.);; 2 MFM Sunalle, Via Ogliastra 10, 08023 Fonni, NU, Italy

**Keywords:** crispy flatbread, germ, bran, middlings, sourdough, consumer acceptance, CATA, JAR, volatile compounds

## Abstract

This study evaluated the use of four wheat milling co-products, i.e., whole wheat germ (WWG), defatted wheat germ (DWG), defatted wheat bran (DWB), and wheat middlings (WM), as ingredients in *carasau* bread, incorporated as powders in baker’s yeast breads (P-series) or after fermentation in sourdough breads (F-series). Therefore, eight formulations were characterized for physicochemical properties and volatile compounds by HS-SPME-GC-MS, while consumer perception was assessed through overall liking, Check-All-That-Apply (CATA), and Just-About-Right (JAR) evaluation. Sourdough-fermented breads showed higher acidity and lower yellowness than baker’s yeast breads formulated with powdered co-products, whereas instrumental texture differed among formulations. Volatile profiles were primarily influenced by co-product type, with sourdough DWG bread showing the greatest modulation of aroma composition. Sensory characterization revealed formulation-specific profiles, showing that comparable levels of overall liking could be associated with different sensory configurations. The most evident differences in overall liking were observed for WWG and WM breads, whereas sourdough DWG and DWB breads showed overall liking distributions comparable with those of the baker’s yeast counterparts. Perceived attribute adequacy also varied among formulations, with baker’s yeast breads generally showing responses closer to the consumer-defined ideal than their sourdough counterparts, although this pattern was not uniform across co-products. Overall, the findings highlight the potential of wheat milling co-products for *carasau* bread production. However, the heterogeneity of consumer responses suggests the need for future studies with larger consumer samples to better define preference patterns and market potential.

## 1. Introduction

The increasing need to improve the sustainability of cereal-based food systems has stimulated interest in the valorization of wheat milling co-products generated alongside flour and semolina, including wheat germ, bran-rich fractions and middlings. Although these fractions are often less extensively used in human nutrition than the main milling streams, being primarily directed to animal feeding, they contain dietary fibre, proteins, minerals, lipids, phenolic compounds, and other bioactive constituents. The term “co-products”, rather than “by-products”, can better reflect the potential of these materials as functional and sustainable food ingredients rather than merely residual processing streams [1,2,3]. Their incorporation into cereal-based foods may therefore contribute to improved resource efficiency and enhance the nutritional and functional value of final products [4,5], although there are few studies regarding the effect of the different co-products in cereal-based foods [2]. Bread is a suitable vehicle for the incorporation of wheat milling co-products because of its widespread consumption and formulation versatility. Co-products are particularly rich in lipids, fibre and ash; they also contain starch and protein in varying amounts, so their composition can differ depending on the milling stage from which they are obtained [2]. However, the inclusion of such co-products containing high levels of lipids (such as germ) or fibre (such as bran) in bread doughs can alter water distribution, dough development, gluten-network structure, gas retention capacity, and final product quality. These ingredients may also influence sensory properties, including colour, texture, mouthfeel, and flavour. In fibre-rich cereal foods, sensory changes are commonly associated with cereal-related notes (i.e., bran-like, cereal-like, toasted) and with bitterness, astringency, dryness, and roughness, which can limit consumer acceptance [6]. The presence of fatty acids with high nutritional value in wheat milling co-products is of particular interest; however, at the same time, lipids in baked goods could lead to rancidity and the development of off-flavours [2,4].

Fermentation has been proposed as a biotechnological strategy for improving the nutritional and sensory characteristics of cereal co-products before their incorporation into foods. The incorporation of fermented wheat milling fractions has consequently been investigated in bread, pasta, and other cereal-based foods [5,7,8,9,10]. Fermentation with selected lactic acid bacteria and yeasts promotes acidification, increases the availability of bioactive compounds, reduces antinutritional factors and generates aroma-active metabolites, with possible positive effects on the sensory quality of the final product [4,7,11].

*Carasau* bread is a traditional, low-moisture, crispy flatbread. Previous work has shown that replacing baker’s yeast with semolina-based liquid sourdough can modify the physical, nutritional and sensory properties of *carasau* bread [12]. Despite growing interest in the fermentation of co-products and their use in food applications, limited information is available on the comparative behaviour of different co-products when incorporated through different leavening strategies, i.e., as unprocessed powder, or via a sourdough system, within crispy flatbreads.

Consumer-oriented sensory methodologies provide valuable information on the relationships between product characteristics and consumer preferences [13,14,15,16]. Among these approaches, the Check-All-That-Apply (CATA) method has gained widespread application in consumer studies due to its simplicity and its ability to efficiently capture descriptive sensory information across multiple samples. In parallel, hedonic acceptance tests are commonly used to evaluate consumer liking [17,18]. Just-About-Right (JAR) scales further enable the assessment of whether the perceived intensity of specific sensory attributes is considered too weak, optimal, or too strong [19]. When combined with penalty analysis, they can be used to identify attributes that negatively affect consumer acceptability [20,21].

HS-SPME-GC-MS is widely used to characterize the volatile profile of bread and to identify flavour-related compounds associated with raw materials, fermentation and baking conditions [22,23]. Although the volatile composition of bread and sourdough has been extensively investigated, most available studies have focused on conventional crumb-structured breads [23,24,25], while comparatively less information is available for *carasau* bread.

To the best of our knowledge, no previous study has jointly compared different wheat milling co-products by comparing their use as is, in baker’s yeast breads, against their incorporation as a sourdough system in *carasau* bread, while simultaneously evaluating physicochemical, volatile, and consumer perception attributes.

This study aimed to evaluate the use of whole wheat germ (WWG), defatted wheat germ (DWG), defatted wheat bran (DWB), and wheat middlings (WM) as ingredients for *carasau* bread formulation. Each co-product was incorporated either as a powder in baker’s yeast formulations or after sourdough fermentation. The resulting breads were characterized for physicochemical properties, volatile profiles, and consumer response through hedonic and consumer-based sensory approaches.

## 2. Materials and Methods

### 2.1. Co-Products, Ingredients, Chemicals and Reagents

Defatted durum wheat germ (DWG), defatted wheat bran (DWB), and semolina used in sourdough fermentations were kindly provided by Casillo Next Gen Food (Corato, Bari, Italy). Whole wheat germ (WWG) and wheat middlings (WM) were provided by Molino Agostini (Massignano, Italy) and by Molino F.lli Brundu (Macomer, Italy), respectively. Semolina used for bread doughs was provided by Brundu S.r.l. (Torralba, Italy).

### 2.2. Sourdough Preparation and Refreshment

Four different sourdoughs were prepared at the Porto Conte Ricerche laboratory, one for each co-product. Each sourdough was obtained by mixing the co-product and semolina at a ratio of 8:2 (*w*/*w*). Water was added according to the hydration capacity of each co-product, as reported in the study conducted by Sanna et al. [26], with the exception of WWG, which was hydrated at a ratio of 1:2.6. The mixtures were then inoculated with selected microbial strains [26]. Four refreshments were carried out by back-slopping, with one refreshment per day. Afterward, the sourdoughs were transferred to the bakery where four additional refreshments were performed before dough preparation and bread making. The microbial strains, the hydration rate of co-products and semolina, and the back-slopping procedure were previously described by Sanna et al. [26]. The acidity values of sourdoughs used for F-bread formulations are reported in Table S1.

### 2.3. Bread-Making Process

*Carasau* bread was produced at the MFM Sunalle bakery factory (Fonni, NU, Italy), according to the traditional two-step baking process, as reported by Fois et al. [27]. The treatments included eight formulations, differing in the co-product type (DWG, DWB, WWG, WM), which were used as untreated powders (P) or fermented with sourdough (F), as reported in Table 1.

The amount of water to be added to the doughs was calculated in order to obtain low-hydrated doughs, as is common for *carasau* bread doughs [12,27], and was based on the bakery’s experience. The dough hydration values and the dough moisture levels are reported in Supplementary Material (Table S1). The amount of sourdough added to each formulation was adjusted to ensure that the corresponding F-dough contained the same amount of co-product as its P-dough counterpart.

P-doughs were leavened by baker’s yeast and F-doughs by sourdough. A small amount of compressed baker’s yeast was added to the sourdough-based formulations to ensure adequate leavening and facilitate handling of the thin dough sheets under bakery processing conditions. The first and the second dough risings lasted approximately 40 and 30 min for the P-doughs, and 180 and 45 min for the F-doughs, respectively.

The first baking was carried out at 550 °C for about 5 s, and toasting at 400 °C for about 5 s, both in an electrical tunnel oven [27]. The four co-products were processed into bread on different days. For the same co-product, the P- and F-bread formulations were baked on the same day.

After the toasting step, the bread was cooled, packaged in a heat-shrinkable plastic film, subsequently transported to the laboratory of Porto Conte Ricerche for analysis, and stored at ambient temperature.

### 2.4. Physicochemical Analysis

Moisture, ash content, pH, and total titratable acidity (TTA) were determined according the methods described by Fois et al. [27]. All analyses were performed on three different breads from each formulation.

The acetic and D/L-lactic acid contents were determined according to the method described by Fois et al. [27], with minor modifications. Briefly, 5.0 g of sample was dispersed in 45 mL of distilled water and magnetically stirred at room temperature for 30 min. The resulting suspension was centrifuged at 14,000× *g* for 10 min at 20 °C. The supernatants were collected and stored at −80 °C until analysis. All extractions and analyses were performed on three different breads from each formulation, and the results were expressed as a percentage (% *w*/*w*).

The texture of *carasau* bread was assessed using a large static deformation test on a TA-XTplus Texture Analyzer (Stable Micro Systems, Godalming, UK) equipped with a 5 kg plugged-in load cell. Measurement of force (Newtons, N) versus time (seconds, s) was recorded using a P/0.25S ¼’’ spherical stainless-steel probe and an HDP/90 heavy-duty platform equipped with a holed plate. The bread sample was placed in the centre of the platform, where the probe penetrated the sample until its complete rupture. The test speed settings were as follows: test speed 0.5 mm/s, distance 10 mm, trigger force 0.05 N and threshold force 0.07 N. Exponent Stable Micro Systems software (v6.1.16) was used for data acquisition and processing. Twenty sheets of bread were analyzed for each bread formulation.

The colour parameters were measured using a CM-700d spectrophotometer (Konica Minolta Inc., Osaka, Japan) with a D65 illuminant and a 10° CIE standard observer. The analysis was conducted on the L* (lightness), a* (redness/greenness), and b* (yellowness/blueness) coordinates. Before the analysis, the spectrophotometer was calibrated using a standard white tile (L* = 93.5, a* = 1.0, b* = 0.8). Analyses were performed on twenty sheets of bread for each formulation, and the results were averaged.

### 2.5. Analysis of Volatile Compounds by HS-SPME-GC-MS

#### 2.5.1. Sample Preparation

For each *carasau* bread formulation, approximately 30 g of bread was randomly collected from a single experimental production batch, and thoroughly homogenized using a laboratory blender. The resulting material was passed through a 1 mm sieve to obtain a homogeneous powder suitable for volatile compound analysis. Three separate vials were then prepared from each homogenized sample. For each vial, 3.00 g of powder was weighed into a 20 mL amber glass headspace vial (Thermo Scientific SureSTART^TM^, Langerwehe, Germany) sealed with an 18 mm magnetic screw cap fitted with a blue silicone/clear PTFE septum (2 mm thickness; Thermo Scientific SureSTART^TM^). An 8 µL aliquot of 2-methyl-3-heptanone (M0693, Tokyo Chemical Industry, Tokyo, Japan), prepared as an 80 mg L^−1^ solution in methanol (Supelco, ≥99%, suitable for GC/MS), was added to each vial as an internal standard, corresponding to a final amount of 640 ng per vial. No sodium chloride or other additives were added prior to headspace extraction.

#### 2.5.2. HS-SPME-GC-MS Conditions

Volatile compounds were extracted by headspace solid-phase microextraction (HS-SPME) using an in-house optimized protocol. The experimental approach was consistent with previously reported headspace SPME methodologies for bread volatile analysis [28]. Extractions were performed using a TriPlus RSH autosampler (Thermo Fisher Scientific, Austin, TX, USA) equipped with a DVB/CAR/PDMS fibre (50/30 µm, 1 cm; Supelco, Bellefonte, PA, USA). Sample vials were incubated at 60 °C for 10 min with intermittent agitation (10 s on/2 s off), followed by headspace extraction at 60 °C for 60 min. After extraction, analytes were thermally desorbed from the fibre in the GC injector at 250 °C for 10 min. Desorption was performed in splitless mode for the first 3 min, followed by split injection at a split flow of 50.0 mL min^−1^ (split ratio 33.3) for the remaining desorption time. Between consecutive analyses, the fibre was conditioned in the dedicated conditioning station at 250 °C for 2 min and additionally pre-conditioned for 2 min before each extraction. Chromatographic analyses were carried out using a TRACE 1310 gas chromatograph coupled to an ISQ LT single quadrupole mass spectrometer (Thermo Fisher Scientific, Austin, TX, USA). Separation was achieved on a Supelco SLB-5MS fused-silica capillary column (60 m × 0.25 mm i.d., 0.25 µm film thickness). Helium (purity 99.999%) was used as the carrier gas at a constant flow rate of 1.0 mL min^−1^. The oven temperature programme was as follows: 60 °C for 5 min; increased to 150 °C at 4 °C min^−1^ and held for 5 min; increased to 250 °C at 5 °C min^−1^; then increased to 300 °C at 20 °C min^−1^ and held for 5 min. Mass spectra were acquired in electron ionization (EI) mode at 70 eV over an m/z range of 50–300. The ion source and transfer line temperatures were maintained at 230 and 260 °C, respectively. Instrument control, data acquisition and automated HS-SPME operations were performed using Xcalibur software v.3.1 (Thermo Fisher Scientific, Austin, TX, USA).

#### 2.5.3. Data Processing

Chromatographic data were processed using the Automated Mass Spectral Deconvolution and Identification System (AMDIS, version 2.72; National Institute of Standards and Technology, Gaithersburg, MD, USA) [29]. A dedicated target library comprising 20 volatile compounds was developed by selecting representative aroma-related compounds reported as relevant contributors to bread aroma in previous literature [23], taking into account the reliability of their chromatographic and mass spectral identification. Compound assignments were supported by comparison with the NIST/EPA/NIH 2014 Mass Spectral Library (NIST 2014, National Institute of Standards and Technology, Gaithersburg, MD, USA), visual inspection of the deconvoluted mass spectra, and verification of chromatographic retention time consistency. Experimental linear retention indices (LRIs) were calculated according to Van den Dool and Kratz [30] using a C8–C20 n-alkane series analyzed under the same chromatographic conditions (column, oven temperature programme and carrier-gas flow). Experimental LRIs were compared with the median experimental RI values reported in the NIST database for semi-standard non-polar stationary phases to further support putative compound assignments (Table S2). For three early-eluting compounds (pentanal, 3-methyl-1-butanol, and 1-pentanol), experimental LRIs could not be calculated because their retention times preceded that of the first n-alkane (C8) in the reference series. Their putative identification was therefore based on mass spectral library matching, examination of deconvoluted spectra, and chromatographic retention-time consistency, without experimental LRI confirmation.

All chromatograms were processed using identical AMDIS settings and the dedicated target library to ensure consistent deconvolution, putative compound identification and peak integration across all samples. AMDIS deconvolution was performed using high shape requirements. Putative compound assignments were accepted only when the deconvoluted spectra fulfilled predefined quality criteria, including a minimum match factor of 80, a minimum of three model peaks and a signal-to-noise ratio of at least 10. For each target compound, the peak area-to-internal standard area ratio (Area/IS) was calculated using 2-methyl-3-heptanone as the internal standard. Normalized Area/IS values for the 20 target VOCs are reported in Table S3 as mean ± standard deviation of three technical replicates. Numerical values were reported only when a compound was consistently detected in all three technical replicates of a formulation; otherwise, the result was indicated as not detected (n.d.). For exploratory multivariate analysis, a CORE VOC dataset comprising 14 compounds was selected from the 20-compound target library. Inclusion in the CORE dataset required consistent detection in all three technical replicates across all eight bread formulations, together with satisfactory analytical repeatability. The remaining six target compounds were not included in the CORE dataset because they did not meet the complete-detection criterion and, in some cases, showed greater variability among technical replicates. This selection was intended to provide a complete and analytically consistent dataset for PCA and heatmap analysis, rather than an exhaustive characterization of the bread volatile fraction. Since absolute or semi-quantitative VOC concentrations were not determined, the normalized Area/IS responses cannot be directly compared with literature odour thresholds expressed as concentrations, and odour activity values (OAVs) could not be calculated.

### 2.6. Consumer Panel and Sensory Evaluation Design

The sensory evaluation involved 62 untrained adult volunteers, a sample size considered sufficient to obtain stable sample and descriptor configurations when products are sufficiently differentiated [31]. All participants received information about the study and provided written informed consent before taking part. The informed-consent documentation was retained by the research institution in accordance with applicable data-protection requirements. Demographic information was collected using a background questionnaire. Participants had a mean age of 42 ± 8 years, and 67.7% were women. More than 80% held a university degree or higher qualification. Regarding occupation, 41.9% were teachers, 27.4% were researchers, and 6.5% were administrative staff. The panel therefore represented a convenience sample predominantly composed of highly educated adults. Accordingly, the sensory findings should be interpreted within the socio-demographic characteristics of the present sample, and should not be considered representative of the general consumer population. The sensory evaluation was conducted over four separate weekly sessions. Each participant evaluated all eight bread formulations. During each session, participants assessed two formulations prepared with the same wheat milling co-product: one containing the untreated powder and the other containing the corresponding fermented ingredient. Immediately before evaluation, each participant received one randomly selected quarter-sheet portion. Because the baked sheets naturally showed areas with different degrees of surface browning and toasted spots, portions were selected at random to avoid preferentially serving areas with lighter or more intense toasting. Each portion was placed in a 30 × 30 cm resealable food-grade plastic bag, identified with a randomly generated three-digit code, and served at ambient temperature. Within each weekly session, the two samples were presented sequentially and monadically in a randomized and balanced order. Approximately half of the participants evaluated the formulation containing the powdered co-product first, whereas the remaining participants received the corresponding fermented formulation first. Only two samples were evaluated in each session to limit sensory fatigue and adaptation. Participants were instructed to rinse their mouths with low-mineral-content water between samples to minimize sensory carry-over residual oral sensations and re-establish a more neutral tasting condition before evaluating the subsequent sample [32,33,34].

#### Sensory Acceptability, CATA and JAR Evaluations

Consumer acceptability was assessed using a nine-point hedonic scale ranging from 1, “dislike extremely”, to 9, “like extremely” [17,35,36].

Before the consumer study, a focus group involving experts in food technology and sensory analysis was conducted to identify and refine sensory attributes relevant for describing *carasau* bread containing wheat milling co-products, while ensuring that the selected terms were understandable to consumers. Focus-group procedures can support the development of appropriate descriptor lists for subsequent consumer-based sensory characterization [37,38]. The resulting terms were used to develop the Check-All-That-Apply (CATA) questionnaire and to select the attributes subsequently evaluated using Just-About-Right scales. The CATA questionnaire and the JAR forms were developed and administered using Google Forms (Google LLC, Mountain View, CA, USA). The hedonic and CATA evaluations were designed and managed using the Smart Sensory Solutions platform (Smart Sensory Solutions S.r.l., Sassari, Italy). For each sample, consumers completed the sensory tasks in the following order: overall liking, CATA, and JAR. Overall liking was assessed first to obtain an initial hedonic response before directing participants’ attention towards specific sensory characteristics. As question order can influence product discrimination and sensory characterization [39,40,41], priority was given to collecting overall liking ratings before the descriptive and attribute-adequacy tasks. After rating overall liking, consumers completed a CATA questionnaire comprising 18 descriptors covering the principal appearance, odour, taste, flavour, and texture/mouthfeel characteristics of *carasau* bread. Participants were instructed to select all descriptors they considered applicable to each sample. Responses were recorded as binary data, with 1 indicating that the descriptor was selected and 0 indicating that it was not selected. The order of the CATA descriptors was randomized and balanced across participants by the Smart Sensory Solutions platform to limit potential positional effects [42,43]. Finally, JAR scales were used to evaluate the perceived adequacy of roughness, hardness, toasted intensity, and sheet thickness. For each attribute, consumers selected one of three response categories: “too low”, “just-about-right”, or “too high”. The central JAR category represented the perceived ideal level of the attribute, whereas the two non-JAR categories indicated deviations below or above that level [20,21,44].

### 2.7. Statistical Analysis

Statistical analysis of physicochemical parameters of *carasau* bread samples was performed using JASP (version 0.98.1) [45]. A one-way ANOVA with post hoc Tukey test (*p* value < 0.05) was completed to identify significant differences among groups. Since the sampled sheets of bread originated from a single baking trial per formulation, the statistical analysis captures the intra-batch variability. When variances were not assumed, a correction using Games–Howell (*p* value < 0.05) post hoc test was applied to the data.

For volatile compound analysis, principal component analysis (PCA) was performed using XLSTAT Statistical and data analysis solution, version 2026 (Lumivero, Denver, CO, USA). Heatmaps were generated from VOC data standardized by Z-score for each compound (column-wise across the eight bread formulations), using conditional colour formatting in Microsoft Excel. PCA was performed using the Pearson correlation matrix, corresponding to standardization of each VOC variable to zero mean and unit variance across formulations.

Sensory analyses were performed using XLSTAT Statistical and data analysis solution, version 2026 (Lumivero, Paris, France). Overall liking scores were analyzed as ordinal repeated-measures data. Since all consumers evaluated all eight *carasau* bread formulations and the score distributions significantly deviated from normality, differences among formulations were assessed using the Friedman test, with consumers considered as blocks and bread formulations as treatments. Corrections for tied ranks were applied, and the overall *p*-value was estimated using 10,000 Monte Carlo simulations. When the Friedman test was significant, pairwise comparisons were performed using the Nemenyi post hoc procedure [46,47]. An internal preference map was generated from individual overall liking scores after centering and scaling consumer responses. Bread formulations were represented as product points, whereas individual consumers were represented according to their preference directions in the factorial space. The first two dimensions were retained for graphical interpretation. Consumer heterogeneity in overall liking patterns was further explored using hierarchical agglomerative clustering. Individual consumer liking scores were centred and scaled prior to clustering, and Euclidean distance with Ward’s agglomeration method was used. The resulting dendrogram is reported in Figure S1.

Binary CATA responses were analyzed separately for each sensory descriptor using Cochran’s Q test to determine whether citation frequencies differed significantly among the bread formulations. When the overall Cochran’s Q test was significant, pairwise comparisons were performed using McNemar’s test with Bonferroni correction. Correspondence analysis was applied to the aggregated CATA citation-frequency table to visualize associations between bread formulations and sensory descriptors. Only descriptors showing significant differences among formulations according to Cochran’s Q test were retained for interpretation, and, unless otherwise specified, statistical significance was set at *p* < 0.05. Bootstrap resampling was used to assess the stability of the product configuration obtained from the correspondence analysis. Principal Coordinate Analysis (PCoA) was performed on the CATA frequency matrix to further explore formulation separation and associations with sensory descriptors.

Overall liking was projected as a supplementary variable to assist interpretation of the associations between sensory characteristics and consumer acceptance. JAR responses were analyzed in conjunction with overall liking scores through penalty analysis. For each attribute and direction of deviation, the proportion of consumers selecting the corresponding non-JAR category was calculated. The mean drop in overall liking was determined as the difference between the mean liking score of consumers who rated the attribute as JAR and that of consumers who rated it as either “too low” or “too high”. A deviation was considered relevant when it involved at least 20% of consumers and was associated with a mean drop greater than 1 liking point. The analysis was used to identify non-JAR deviations associated with lower overall liking and to prioritize attributes for potential product optimization. Direction-specific weighted penalties were calculated according to the following equation: weighted penalty = p(non-JAR) × mean drop, where p(non-JAR) is the proportion of consumers selecting the corresponding non-JAR response, following the weighted-penalty approach described by Popper [48] and Rothman and Parker [20]. These values were reported as Total Penalty Scores (TPS) and interpreted descriptively as prioritization indices rather than as tests of statistical significance (Figure S2; Table S4).

## 3. Results

### 3.1. Physicochemical Properties of Bread Samples

The physicochemical properties of *carasau* bread samples are reported in Table 2. The moisture content of the samples ranged between 5.56% and 7.06%. These values are consistent with those generally reported for *carasau* bread [27,49]. The ash content remained around 3% (d.b.) in all samples, with only minor differences. This result is consistent with the dough formulation, as the same amount of co-product and sea salt was used in all dough formulations. Regarding acidity, expressed as pH and total titratable acidity (TTA), all sourdough breads showed a trend toward lower pH values and higher TTA values than the corresponding baker’s yeast leavened breads prepared with the powdered co-products. In the former, pH decreased, reaching the lowest value (4.97) in the WM-F sample, whereas in the latter it ranged from 6.17 to 6.30. Similarly, all sourdough breads exhibited a clear trend toward increased titratable acidity, with values ranging from 6.50 to 11.37 mL of 0.1 N NaOH, whereas baker’s yeast leavened breads prepared with powdered co-products showed TTA values ranging from 4.12 to 5.10 mL of 0.1 N NaOH. Despite the acidification induced by sourdough microorganisms, the pH values remained slightly higher than the median value of 4.5 reported by De Bondt et al. [50] for sourdough breads prepared with negligible amounts of baker’s yeast (<0.2%). The extent of the increase in TTA depended on the type of co-product used, with the highest value observed in the WM-F sample. These findings are consistent with previous studies reporting that sourdough fermentation promotes the production of organic acids, mainly lactic and acetic acids, resulting in a decrease in pH and an increase in titratable acidity [51].

Regarding organic acids, lactic acid was not detected in baker’s yeast breads, as expected, whereas it was present in sourdough breads, confirming the contribution of the fermentation process to the acidification of the final products. Among sourdough samples, WM-F exhibited the highest lactic acid concentration, followed by DWG-F and DWB-F, whereas WWG-F showed the lowest value. Acetic acid was detected at low residual levels in all breads, suggesting that it had a limited contribution to the acid profile of the final baked products, which may be related to its relatively high volatility and partial evaporation during baking.

Bread colour, evaluated in the CIELab colour space (L*, a*, b*), showed limited differences among formulations. For the L* (lightness) coordinate, lower values were observed in breads containing whole wheat germ (WWG-P and WWG-F), indicating that these samples were slightly darker than the other bread samples. Regarding a* (redness), it showed almost no variation among baker’s yeast leavened breads, and WM-P exhibited the lowest value. When baker’s yeast leavened breads were compared with their sourdough counterparts, only F-DWB breads exhibited a slightly higher a* value than their corresponding P-breads. For the b* (yellowness) coordinate, the DWG-P exhibited the highest value among the baker’s yeast breads formulated with powdered co-products. Comparison between baker’s yeast breads and their sourdough counterparts revealed a trend toward increased b* values in the P-breads than in the F-breads, whereas no differences were detected for DWB. Variations in the coordinates were more evident among the co-products than between leavening systems: comparisons between baker’s yeast breads formulated with powdered co-products and their corresponding sourdough counterparts, revealed variations only for the b* (yellowness) coordinate, indicating that sourdough fermentation had a limited influence on the colour characteristics of the final product.

Five mechanical parameters, describing the texture of the bread sample, were derived from the force–displacement curves obtained during the compression test: first break force, maximum break force, work of deformation, number of force peaks, and gradient (Table 3). The first break force, corresponding to the first significant force peak detected during compression, represents the force required to initiate the breakdown of the bread structure and evokes the mechanical sensation perceived during the first bite during consumption. The maximum break force, defined as the highest force peak, reached immediately before complete structural failure, represents the hardness of the bread. The work of deformation, estimated as the area under the force–displacement curve between the onset of fracture and the maximum break force, represents the mechanical energy required to propagate structural failure and provides an indication of the toughness of the bread; higher values indicate resistance to fracture propagation. The number of force peaks occurring after the onset of fracture, until the end of the test, provides an indication of crispiness. Each peak corresponds to an individual fracture occurring during compression. The gradient, calculated as the slope of the linear portion of the force–displacement curve from the initial contact point to the maximum break force, provides an indication of stiffness.

The first break force, maximum break force, gradient, and work of deformation were different among breads, whereas no differences were observed in the number of force peaks. The first break force, which reflects the resistance to the onset of structural failure, was similar among the breads, except for WM ones, both P- and F-, which showed the lowest values. In the same way, the maximum break force, corresponding to the highest force sustained before complete structural collapse, was greater in WWG breads, whereas the remaining formulations showed similar intermediate values. Differences among formulations were also evident in the work of deformation. WWG and WM samples exhibited the highest energy requirement for fracture propagation, whereas DWG and DWB samples showed the lowest value, suggesting a less mechanically resistant and therefore a weaker structure, with a reduced ability to absorb energy before failure, and also suggesting that the lipid fraction may play a key role in influencing this textural parameter. The gradient ranged from 0.48 to 1.17 N/s. WWG breads, both P- and F-, exhibited the highest stiffness, whereas WM breads, both P- and F-, showed the lowest gradient, indicating a softer and less rigid structure. The other four formulations presented intermediate stiffness values. In contrast, the number of force peaks, commonly associated with instrumental crispness, did not appear to differ among breads. This finding suggests that, despite differences in structural resistance and stiffness, the fracture mechanism during compression remained comparable across all bread samples. It is worth mentioning that the gradient and the work of deformation describe two distinct aspects of the mechanical behaviour of crisp breads similar to *carasau*. The gradient of the force–displacement curve is related to stiffness, reflecting the rate of force increase during the deformation phase before the complete fracture. Conversely, the area under the force–displacement curve represents the work of deformation (toughness), i.e., the amount of mechanical energy required to propagate structural failure and complete the fracture. The combination of these parameters provides complementary information on the textural characteristics of the bread samples, since they represent different mechanical properties: stiffness, hardness and toughness do not necessarily coexist in a food matrix. For example, WWG-P simultaneously exhibited the highest gradient, work of deformation and maximum break force, indicating a stiff, hard and tough structure, capable of both resisting deformation and requiring a large amount of energy to fracture. On the other hand, WM-F showed the lowest gradient and a high work of deformation, therefore suggesting a structure with lower initial stiffness but a greater ability to absorb mechanical energy before failure, behaving as a more deformable yet resistant matrix. Conversely, DWG-F showed both a high gradient and the lowest work of deformation values, indicating a resistant structure, characterized by great stiffness but a limited energy absorption capacity during the whole fracture. These results highlight that hardness, stiffness, and toughness should not be considered interchangeable descriptors of crisp bread texture, and this can be the reason why the simultaneous evaluation of all these parameters provides a more comprehensive description of the mechanical behaviour of crisp bread structures. Interestingly, breads did not differ in their crispness, as measured by the number of force peaks, but it is worth noting that there was a significant correlation (*p* < 0.01) between crispness and work of deformation (r = 0.879, *n* = 8), indicating that samples characterized by a more complex fracture pattern undergo greater energy dissipation during the compression. Overall, the differences between P- and F-breads were not consistent, whereas the type of co-product appeared to have a greater influence than the leavening and processing technology.

### 3.2. Volatile Compounds by HS-SPME-GC-MS

#### 3.2.1. Overall Volatile Fingerprint of *Carasau* Formulations

To obtain an overview of the volatile fingerprint of the eight *carasau* bread formulations, the selected CORE volatile compounds were visualized using a heatmap based on Z-score standardized data (Figure 1). Overall, the volatile profile was primarily associated with the type of wheat milling co-product, whereas differences between the corresponding powdered/baker’s yeast and fermented/sourdough formulations were generally less pronounced. Within the investigated samples, fermented defatted wheat germ (DWG-F) displayed the most distinct volatile fingerprint, characterized by a coordinated increase in several volatile compounds. In contrast, whole wheat germ (WWG-P/WWG-F), defatted wheat bran (DWB-P/DWB-F), and wheat middlings (WM-P/WM-F) showed comparatively smaller differences between fermented and non-fermented samples, indicating that differences between powdered/baker’s yeast and fermented/sourdough formulations depended on the type of wheat milling co-product.

Principal component analysis (PCA; Figure 2A) further characterized the relationships among bread formulations based on their CORE volatile profiles. The first principal component (PC1) explained 82.84% of the total variance and showed a marked separation of DWG-F from the other formulations within the investigated dataset, whereas the second principal component (PC2) accounted for an additional 9.08% of the variance. Together, PC1 and PC2 explained 91.92% of the total variance. Examination of the PCA loading plot (Figure 2B) indicated that the separation along PC1 reflected the coordinated contribution of several volatile compounds rather than the dominance of a single marker, whereas octanal contributed most strongly to the variability explained by PC2.

These multivariate analyses consistently indicated that differences in volatile fingerprint between powdered/baker’s yeast and fermented/sourdough formulations were strongly dependent on the specific wheat milling co-product used.

#### 3.2.2. Co-Product-Dependent Modulation of Individual Volatile Compounds

To further investigate differences in individual volatile compounds between powdered/baker’s yeast and fermented/sourdough formulations, the relative abundance of the CORE VOCs was compared within each wheat milling co-product using fold change (FC), calculated from internal-standard-normalized GC-MS peak areas as the ratio between the fermented and corresponding non-fermented (powdered) formulations (Table 4). Overall, the magnitude and direction of the differences between corresponding formulations were strongly co-product-dependent.

Within the investigated dataset, the largest differences in volatile profiles were observed for DWG when comparing the powdered/baker’s yeast and fermented/sourdough-based formulations. Compared with DWG-P, DWG-F exhibited coordinated increases in several volatile compounds, including aldehydes, alcohols and furans. In the PCA score plot, DWG-F appeared separated from the other formulations within the investigated dataset, indicating a distinctive volatile profile under the specific experimental conditions examined.

In contrast, WWG, DWB, and WM generally showed less pronounced and more compound-specific differences between the corresponding P and F formulations. Depending on the formulation, individual volatile compounds increased, decreased or remained essentially unchanged, further supporting a co-product-dependent pattern rather than a general response common to all formulations.

Among the CORE VOCs, (Z)-2-heptenal showed the largest increase in DWG-F (FC = 12.91). Substantial increases were also observed for hexanal, 2-pentylfuran, (E,E)-2,4-nonadienal, heptanal and 1-octen-3-ol. In contrast, octanal displayed a more formulation-specific behaviour. Phenylethyl alcohol also showed markedly different fold changes among co-product types, decreasing in WWG, increasing in DWG and DWB, and remaining essentially unchanged in WM.

### 3.3. Sensory Evaluation

#### 3.3.1. Overall Liking and Internal Preference Mapping

The distribution of overall liking scores for the different *carasau* breads is summarized in Figure 3: a significant effect was detected (Q = 182.163, *p* < 0.0001). All breads showed median overall liking scores around 7–8 on the nine-point hedonic scale, except WWG-F, which showed the lowest median overall liking score (approximately 6), indicating a generally favourable consumer response. Generally, P-breads showed a more concentrated distribution of liking scores than F-breads, with the unique exception of the DWG samples, which displayed liking distributions comparable to those of their corresponding baker’s yeast counterparts. In addition, for WWG-P and DWB-P the median coincided with the first quartile, whereas for WM-P it coincided with the third quartile, indicating a concentration of responses around the median value. All F-breads showed greater dispersion towards lower scores than the corresponding P-breads, indicating that the responses were more variable at the lower end of the hedonic scale, in the F-breads. Among all samples, WM-F exhibited the widest distribution of scores, indicating the greatest variability in individual consumer responses.

The internal preference map (Figure 4) accounted for 71.96% of the total variability in consumer preferences, with F1 and F2 explaining 54.65% and 17.31%, respectively. All consumer preference vectors had positive coordinates on F1, indicating a common orientation along the main preference dimension. WWG-F was positioned on the negative side of F1, opposite to the consumer preference vectors, consistent with its lower overall liking scores (Figure 3). WM-F was also located on the negative side of F1, although it was separated from WWG-F along F2. In contrast, WWG-P, DWB-P, DWB-F, and WM-P were positioned on the positive side of F1, and closely aligned with the overall preference direction, indicating greater consumer acceptance, whereas DWG-P and DWG-F were located closer to the origin on the positive side of F1. Hierarchical clustering based on mean-centred individual overall liking scores identified three groups with different hedonic response patterns (Figure S1).

#### 3.3.2. CATA Sensory Characterization and Correspondence Analysis

Significant differences in CATA citation frequencies were observed among the eight *carasau* breads for several sensory descriptors (Table 5). Among the baker’s yeast breads, WWG-P had the highest citation proportion for wholegrain flavour (0.677) and was also frequently associated with savoury taste (0.855), toasted flavour (0.855), golden colour (0.855), cereal odour (0.613), and glass-like/sharp crispness (0.645). Bitter taste was not selected for this formulation. DWG-P was mainly associated with golden colour (0.887), uniform colour (0.710), semolina odour (0.645), savoury taste (0.726), and toasted flavour (0.758). WM-P showed the highest citation proportion for uniform colour (0.887) and comparatively low proportions for astringent/dryness (0.161), sandy mouthfeel (0.081), and slight acidity (0.113). DWB-P showed the highest citation proportion for fragile/easily breakable (0.806) and the lowest citation proportion for glass-like/sharp crispness (0.177). Among the sourdough breads, WWG-F had the highest citation proportions for astringent/dryness (0.677) and stale flavour (0.242), whereas bitter taste was rarely cited (0.032). DWG-F retained relatively high citation proportions for toasted flavour (0.839) and savoury taste (0.806). DWB-F showed the highest citation proportions for toasted flavour (0.935), burnt spots (0.774), and bitter taste (0.323), and was also relatively frequently associated with wholegrain flavour (0.452). WM-F was primarily distinguished by slight acidity (0.758), sandy mouthfeel (0.355), and astringent/dryness (0.403). Citation proportions for slight acidity differed markedly among breads. WM-F showed the highest citation proportion for this descriptor (0.758), while DWB-F and DWG-F showed intermediate proportions (0.516 and 0.452, respectively). In contrast, slight acidity was infrequently associated with WWG-F (0.145), and its citation proportion did not differ significantly from that of WWG-P. Stale flavour also significantly discriminated among breads and was selected for WWG-F by 15 of the 62 consumers (0.242), whereas it was rarely or never selected for the other breads.

Pairwise comparisons among samples showed significant changes in CATA citation frequencies. Compared with WWG-P, WWG-F showed significantly lower citation proportions for golden colour, toasted flavour, wholegrain flavour, savoury taste, and glass-like/sharp crispness, together with significantly higher proportions for astringent/dryness and stale flavour. DWG-F differed from DWG-P through significantly higher citation proportions for burnt spots and slight acidity. Compared with DWB-P, DWB-F showed significantly higher citation proportions for burnt spots, toasted flavour, bitter taste, slight acidity, and glass-like/sharp crispness, together with significantly lower proportions for uniform colour and fragile/easily breakable. WM-F showed significantly higher citation proportions for semolina odour, slight acidity, sandy mouthfeel, and astringent/dryness, and a significantly lower citation proportion for uniform colour than WM-P.

The bootstrap confidence ellipse plot (Figure 5A) showed differences in the CATA-based configuration of the *carasau* breads. The first two dimensions accounted for 57.41% of the total inertia, with F1 explaining 30.54% and F2 26.87%. WWG-F was positioned on the positive side of F2 and showed limited overlap with the confidence regions of most other breads. DWB-F occupied the negative side of F1, with its confidence region partially overlapping that of WM-F. WM-P was located on the positive side of F1 and the negative side of F2, whereas WM-F occupied the negative-F1/positive-F2 region. DWB-P was positioned on the positive side of F1 and showed a relatively broad confidence ellipse. DWG-P, DWG-F, and WWG-P were located in the central-to-lower part of the configuration and showed partially overlapping confidence regions. Overall, WWG-F and DWB-F occupied relatively distinct regions of the first two-dimensional space, whereas greater overlap was observed among several of the remaining breads.

The biplot in Figure 5B showed the associations among the *carasau* breads, discriminating CATA descriptors, with overall liking projected as a supplementary variable. WWG-F was positioned in the upper part of the plot, in the direction of astringent/dryness, consistent with its high citation proportion for this descriptor. DWB-F was located on the negative side of F1, in the general direction of burnt spots, glass-like/sharp crispness, and toasted flavour, consistent with its high citation proportions for toasted flavour and burnt spots. WM-F occupied the upper-left region of the map, in the same general direction as sandy mouthfeel and astringent/dryness. WWG-P was positioned in the lower-left region, close to toasted flavour, cereal odour, golden colour, and savoury taste. DWG-F was located in the same general region, in the direction of golden colour, cereal odour, and savoury taste, whereas DWG-P occupied a more central position. WM-P and DWB-P were located on the positive side of F1; WM-P occupied the lower-right region, whereas DWB-P was positioned closer to uniform colour. The overall liking vector was oriented towards the lower-central region of the map, broadly in the direction of savoury taste and golden colour and opposite to astringent/dryness and sandy mouthfeel.

#### 3.3.3. JAR Profiles of Perceived Attribute Adequacy

The JAR profiles (Figure 6) showed differences in the perceived adequacy of roughness, hardness, toasted intensity, and thickness across breads. Among the baker’s yeast breads formulated with powdered co-products (Figure 6A), WM-P showed the highest overall JAR proportions, with 72.58% for roughness, 79.03% for hardness, 75.81% for toasted intensity, and 80.65% for thickness. DWB-P showed similarly high JAR proportions for roughness (72.58%) and hardness (66.13%), but toasted intensity and thickness were perceived as too low by 40.32% and 33.87% of consumers, respectively. For DWG-P, JAR proportions ranged from 56.45% for toasted intensity to 67.74% for thickness, while toasted intensity was considered too low by 30.65% of consumers, whereas hardness and thickness were judged too high by 24.19% and 25.81% of consumers, respectively. WWG-P also showed predominantly JAR responses, but roughness and thickness were perceived as too high by 29.03% and 32.26% of consumers, respectively.

Among the sourdough breads (Figure 6B), DWG-F showed relatively high JAR proportions, particularly for hardness (75.81%) and thickness (69.35%), although roughness, toasted intensity, and thickness were judged too high by approximately 27–29% of consumers. DWB-F showed 50% JAR responses for roughness, toasted intensity, and thickness. Excessive toasted intensity was its most frequent deviation, reported by 43.55% of consumers, while roughness was considered too high by 35.48%. WWG-F showed the lowest JAR proportions among the fermented formulations for hardness (29.03%) and toasted intensity (40.32%). Hardness generated responses in both directions, with 29.03% of consumers selecting too low and 41.94% too high. Toasted intensity showed a clearer directional deviation, with 48.39% of consumers considering it too low. Thickness and roughness were also rated as too high by 37.10% of consumers in both cases. WM-F showed comparatively high JAR proportions for hardness (72.58%), toasted intensity (66.13%), and thickness (61.29%); however, roughness was judged too high by 35.48% of consumers.

Overall, WM-P showed the highest proportion of JAR responses across the evaluated attributes. These response frequencies identify the most common deviations from the ideal level but do not establish whether they were associated with reduced overall liking; their hedonic relevance was therefore assessed through penalty analysis.

#### 3.3.4. Penalty Analysis of JAR Deviations

The penalty analysis for the baker’s yeast breads formulated with powdered co-products is shown in Figure 7A. Deviations from the JAR level were considered relevant when they involved at least 20% of consumers and were associated with a mean drop in overall liking greater than 1 point. Only three deviations met both criteria. For DWG-P, excessive hardness and thickness involved approximately 24–26% of consumers and were associated with mean drops of about 2.3 and 2.1 liking points, respectively. For WWG-P, insufficient toasted intensity was reported by approximately 26% of consumers and was associated with a mean drop of about 1.4 points. No deviation identified for DWB-P or WM-P exceeded both predefined thresholds. In summary, the relevant penalties for the untreated formulations concerned excessive hardness and thickness in DWG-P and insufficient toasted intensity in WWG-P.

The penalty analysis for formulations containing fermented co-products is shown in Figure 7B. For WWG-F, insufficient toasted intensity was the most frequent relevant deviation, involving almost half of consumers and being associated with a mean drop of approximately 1.4 liking points. Insufficient hardness also met both predefined criteria, involving approximately 29% of consumers and being associated with a mean drop of approximately 1.6 liking points. DWG-F showed relevant penalties for excessive roughness, toasted intensity, and thickness, associated with mean drops of approximately 2.9, 2.8, and 1.6 liking points, respectively. For DWB-F, excessive toasted intensity was the most frequent relevant deviation, involving more than 40% of consumers and being associated with a mean drop of approximately 2.1 points; excessive hardness and thickness also exceeded both predefined thresholds. WM-F showed the largest number of relevant deviations, with excessive roughness, hardness, toasted intensity, and thickness all involving more than 20% of consumers and being associated with mean drops of approximately 2.7–3.1 points. Excessive roughness was the most frequently reported deviation, whereas excessive hardness showed the greatest mean drop. Overall, WWG-F was mainly associated with insufficient toasted intensity, whereas the relevant penalties for DWG-F, DWB-F, and WM-F were predominantly related to attributes perceived as excessive. The direction-specific Total Penalty Scores (TPS) summarized the prevalence and hedonic impact of JAR deviations (Figure S2). Detailed results, including the proportion of consumers in each non-JAR category, the corresponding mean drop in overall liking, the TPS, and the criterion used to identify relevant deviations, are reported in Table S4. TPS values were predominantly associated with the “too-low” direction in the WWG formulations, particularly for toasted intensity in WWG-F, whereas DWG, DWB, and WM breads showed mainly “too-high” penalties. The highest and most broadly distributed TPS values were observed for WM-F, while DWB-P and WM-P showed comparatively limited penalty profiles.

## 4. Discussion

The consumer responses to baker’s yeast breads formulated with powdered co-products or their sourdough counterparts varied according to the specific co-product, indicating that the effect of baker’s yeast or sourdough technology cannot be generalized across the different matrices. Previous studies have demonstrated the potential of microbial fermentation as a strategy for upgrading cereal milling co-products into value-added food ingredients [4,5,10]. In the present study, however, sourdough technology did not produce a uniform improvement or deterioration in consumer acceptance. DWG-F and DWB-F showed overall liking distributions broadly comparable with those of their baker’s yeast counterparts, whereas the greatest changes were observed for WWG and WM. WWG-F showed a lower median liking score and a broader distribution than WWG-P, whereas WM-F differed from WM-P mainly in the greater dispersion of liking scores. Thus, even though the sourdoughs were obtained using the same microorganisms and fermentation procedure, their incorporation into bread resulted in different hedonic responses depending on the starting matrix. This formulation-specific behaviour is consistent with evidence that the sensory properties of cereal foods containing wholegrain or bran-rich fractions depend strongly on the cereal fraction incorporated and on processing conditions [6]. Differences in fibre, proteins, residual lipids, minerals, phenolic compounds and endogenous enzymes may affect the response of each matrix to fermentation and the subsequent breadmaking process, helping to explain why the same technological treatment does not necessarily produce comparable sensory outcomes across different co-products [3,4,5,52].

The combined liking and CATA results further showed that comparable levels of consumer acceptance could correspond to markedly different sensory configurations. WWG-P was more frequently associated with wholegrain flavour, savoury taste, toasted flavour, golden colour and glass-like/sharp crispness than WWG-F, and also showed a higher median liking score. Conversely, the lower acceptance of WWG-F occurred together with higher citation frequencies for astringent/dryness and stale flavour. The latter should be interpreted strictly as a consumer-reported sensory descriptor, rather than as direct evidence of product deterioration. Negative sensory descriptors, such as stale flavour, could be the consequence of the interactions between the sourdough metabolism, a substrate with a high lipid content, and baking at high temperature. Astringency, together with bitterness and dryness, has been identified as an important sensory dimension in wholegrain cereal products and may contribute to differences in their consumer acceptance [6,53,54]. On the other hand, in DWG breads, the use of sourdough modified the sensory configuration without producing an evident variation in overall liking. DWG-F showed higher citation frequencies for slight acidity, burnt spots, toasted flavour and savoury taste than DWG-P, while maintaining a broadly comparable liking distribution. The greater perception of slight acidity is coherent with the physicochemical results, since sourdough fermentation resulted in lower pH and higher titratable acidity and organic acid concentrations, as expected. It is also consistent with Perri et al. [5], who reported a perceptible acidic taste in bread containing fermented defatted durum wheat germ. Thus, in these breads, the increased acidic and toasted sensory notes associated with the use of sourdough technology were not accompanied by a corresponding loss of consumer acceptance.

A comparable dissociation between sensory differentiation and hedonic response was observed for DWB. DWB-F was more frequently described as having burnt spots, toasted flavour, bitter taste, slight acidity and glass-like/sharp crispness than DWB-P, despite the fact that the two breads showed similar overall liking distributions. The relatively high citation frequency of bitter taste in DWB-F, together with toasted flavour and burnt spots, may be partly related to compounds generated during high-temperature baking. Maillard-derived compounds formed in bread crust have previously been associated with bitterness perception [55,56]. In *carasau* bread, these reactions may be particularly relevant because the production process includes an intense final toasting step at approximately 400 °C. Nevertheless, the present results also indicate that bitterness cannot be considered intrinsically detrimental to acceptance, since its higher citation frequency in DWB-F was not accompanied by lower liking.

WM displayed a further sensory pattern. Compared with WM-P, WM-F showed higher citation frequencies for semolina odour, slight acidity, sandy mouthfeel and astringent/dryness, while WM-P was more frequently associated with uniform colour. The perception of astringency, sandy texture and roughness is quite common in high-fibre breads [6], likely amplified by sourdough fermentation. The higher citation of slight acidity in WM-F is consistent with its physicochemical characteristics, including its lower pH and higher titratable acidity and lactic acid concentration. At the same time, WM-F showed a broader distribution of liking scores than WM-P, indicating greater variability in individual consumer responses rather than a uniform negative effect of fermentation. This result suggests that the sensory changes induced by the use of sourdough in this matrix may have been acceptable to some consumers but less so for others.

The JAR results provided complementary information on the adequacy of selected sensory attributes. Overall, baker’s yeast breads tended to show profiles closer to the ideal levels than their sourdough counterparts. The contrast was particularly evident for DWB and WM: no deviations fulfilled both penalty-analysis criteria in DWB-P or WM-P, whereas relevant deviations were identified in the corresponding sourdough breads. These findings suggest that sourdough technology may have altered the balance of specific texture or flavour attributes even when overall liking was not substantially reduced. Penalty analysis should, however, be interpreted as a diagnostic rather than causal tool. Mean drops describe associations between non-JAR responses and liking among consumers selecting a particular response category and do not demonstrate that modifying an individual attribute would necessarily produce a corresponding increase in acceptance [44,57,58].

The sensory texture results can also be considered alongside the instrumental measurements. Differences in formulation and processing technology mainly affected bread mechanical strength and stiffness, whereas the number of force peaks did not differ significantly among samples. This suggests that the resistance to deformation varied according to the co-product and processing approach, while the overall fracture behaviour associated with crispness remained relatively comparable across breads. Therefore, instrumental texture and consumer perception appear to provide complementary information: changes in mechanical properties did not necessarily translate into a uniform perception of inadequate crispness or into lower liking, while JAR analysis identified bread-specific deviations in attributes such as hardness, roughness, thickness and toasted intensity.

Instrumental colour differences were likewise driven more by the type of co-product than by the comparison between leavening strategy. Nevertheless, the use of sourdough was associated with lower b* values, indicating a reduction in the yellow component of the bread colour. Similar decreases in b* have been reported for sourdough breads compared with breads leavened with baker’s yeast [59]. Codină et al. [60] observed progressively lower b* values with increasing sourdough addition. Changes in cereal pigments, particularly carotenoids, during sourdough fermentation may contribute to this behaviour [61], although the final colour of *carasau* bread is also strongly influenced by the high-temperature toasting step and the associated Maillard and caramelization reactions [62]. The instrumental results are therefore consistent with the visually different sensory configurations observed among breads, such as the greater citation of golden colour for WWG-P or uniform colour for WM-P, although a direct relationship between CIELab coordinates and these consumer-selected descriptors cannot be established from the present data.

As expected, the different leavening systems also influenced bread acidity, with sourdough breads showing lower pH and higher titratable acidity and organic acid concentrations [63]. The predominance of lactic acid is consistent with lactic acid fermentation, whereas the small amounts of acetic acid detected may reflect several factors associated with the raw material and fermentation process. Previous work has also indicated that grain treatments before milling may contribute to the organic acid profile of the resulting cereal fractions [26]. Importantly, the sensory relevance of the increased acidity was dependent on the type of co-product: slight acidity was more frequently cited in several sourdough breads, particularly DWG-F and WM-F, but this did not invariably correspond to reduced liking.

The volatile compound profile provided an additional level of interpretation. Within the investigated dataset, differences in volatile profiles were observed between the powdered/baker’s yeast and fermented/sourdough-based formulations, with DWG-F exhibiting a particularly distinct profile. This behaviour may reflect differences in substrate composition and aroma-precursor availability, which can influence microbial metabolism and subsequent volatile formation [5,23]. Fermentation of defatted wheat germ has previously been associated with extensive proteolysis and increased free amino acid availability [5], which could contribute to the broader modulation observed in DWG-F. Lipid-related pathways may also have contributed to the distinctive response of DWG-F. Wheat germ contains endogenous lipolytic and oxidative enzymes, and higher extractable lipoxygenase activity has been reported in defatted than in raw wheat germ [64]. However, sourdough fermentation should not necessarily be expected to increase lipolytic activity per se, since fermentation of wheat germ has been reported to markedly decrease endogenous lipase activity and to limit the accumulation of lipid-oxidation-derived volatiles during storage [11]. Furthermore, since VOCs were measured in the final baked product, the observed profiles should be interpreted as the combined outcome of biochemical differences associated with co-product type and leavening strategy, together with thermal reactions occurring during baking. Consistent with this interpretation, the coordinated increases observed in DWG-F for hexanal, (Z)-2-heptenal, 1-octen-3-ol, 2-pentylfuran and (E,E)-2,4-nonadienal are compatible with an increased contribution of lipid-derived pathways, since these compounds or their precursors can originate from the oxidative degradation of unsaturated fatty acids, although their final abundance may also be influenced by thermal processing [23,65]. Phenylethyl alcohol, commonly associated with microbial amino acid metabolism, increased predominantly in DWG-F [23]. Importantly, the magnitude of volatile modulation did not directly parallel either sensory differentiation or consumer acceptance. Despite exhibiting a distinctive volatile fingerprint within the investigated dataset, DWG-F maintained comparatively favourable consumer responses. Volatile profiling should therefore be regarded as complementary to sensory evaluation rather than as a direct predictor of sensory quality. An important limitation of the present VOC analysis is that each bread formulation was represented by a single production batch, with three technical replicates used to assess analytical repeatability. Consequently, the observed differences in volatile profiles and the separation patterns identified by PCA should be interpreted as exploratory findings specific to the investigated samples and processing conditions. Independent production replicates would be required to assess between-batch variability and establish the reproducibility of these patterns. Furthermore, the multivariate analysis was restricted to a selected set of 14 consistently detected aroma-related VOCs and does not represent the complete volatile composition of *carasau* bread.

Taken together, the results indicate that the technological and sensory consequences of incorporating wheat milling co-products as sourdough into *carasau* bread are strongly matrix-dependent. Fermentation generated distinct modifications in acidity, texture, colour, volatile composition and sensory perception, but these changes did not translate into a uniform hedonic response. In particular, sensory configurations including acidity, bitterness or toasted notes were compatible with comparatively favourable acceptance in some sourdough breads, whereas astringency/dryness and stale flavour were associated with less favourable responses in others. The heterogeneity observed in individual liking responses also suggests that future studies with larger consumer samples could investigate preference heterogeneity by combining liking-based consumer segmentation with CATA responses, with the aim of identifying sensory attributes associated with the preferences of different consumer groups [66,67]. In addition, the evolution of specific volatile and sensory signatures during storage, depending on the co-product and the leavening strategy, warrants further investigation, particularly in relation to lipid oxidation, loss of freshness and stale-flavour development, for which HS-SPME-GC-MS can provide a useful complementary tool [28].

## 5. Conclusions

The present study showed that the incorporation of wheat milling co-products, as powders in baker’s yeast breads or after fermentation in sourdough breads, into *carasau* bread, resulted in distinct physicochemical, volatile, sensory, and hedonic profiles, depending on both the specific co-product and the leavening strategy. Physicochemical, textural, and volatile analyses provided complementary information on such effects, whereas consumer perception represented the integrated response to multiple sensory cues rather than to individual analytical parameters considered in isolation. The combined sensory analyses further showed that comparable levels of liking could be associated with different sensory configurations and that the aspects requiring optimization differed among the investigated breads. The use of sourdough did not exert a uniform effect on consumer acceptance, and may represent an effective valorization strategy for some co-products, whereas for others direct incorporation in powdered form and baker’s yeast leavening was associated with similarly favourable, and, in some cases, more consistent consumer responses. These results reinforce the potential of wheat milling co-products as ingredients for cereal-based foods, while also showing that their performance depends on both the specific matrix and the treatment applied. Overall, the findings therefore highlight the need for tailored strategies, defined according to the specific characteristics of each co-product and capable of integrating technological performance, sensory quality, and consumer acceptance. From an industrial perspective, direct incorporation of untreated wheat milling co-products may represent a simpler and potentially more economically attractive valorization strategy, as it avoids the additional processing required for sourdough fermentation. However, sourdough fermentation can generate more complex and diverse sensory and volatile compound profiles; though effectively leveraging them requires optimizing the formulation and process conditions. Future studies should assess the sensory stability of the most promising formulations during storage and, using larger consumer samples, further investigate preference heterogeneity by combining liking-based consumer segmentation with CATA responses to identify sensory attributes associated with the liking patterns of different consumer groups.

## Figures and Tables

**Figure 1:**
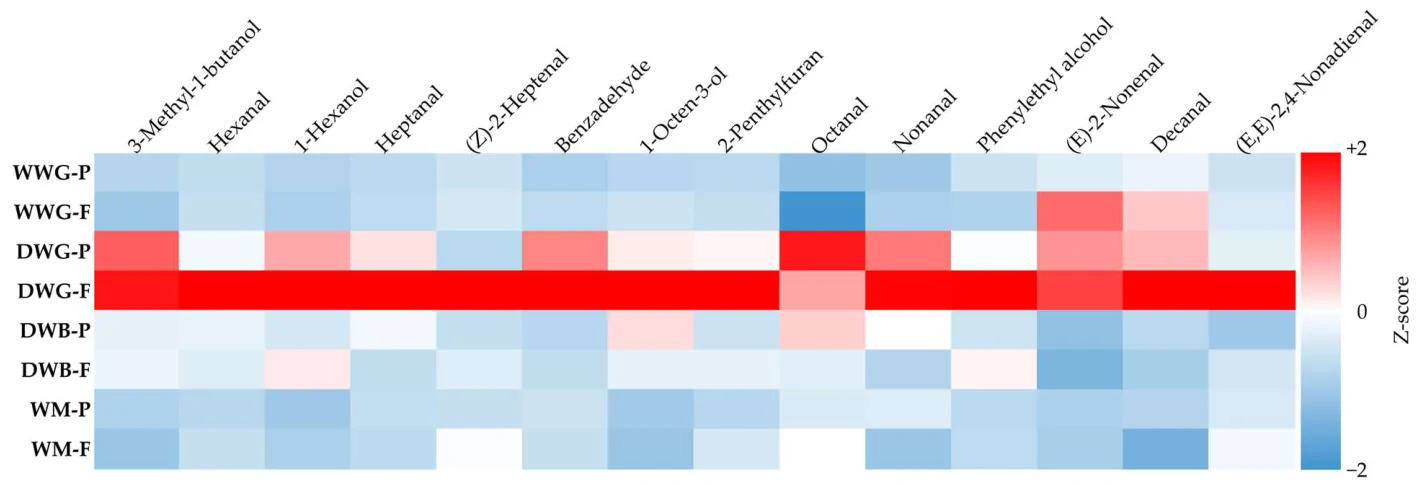
Heatmap of the CORE volatile organic compounds (VOCs) detected in *carasau* bread formulations containing wheat milling co-products, incorporated as powders in baker’s yeast breads (P-series) or after fermentation in sourdough breads (F-series). Cell colours represent the Z-score standardized values calculated separately for each VOC across the eight bread formulations. Rows represent the eight formulations, whereas columns correspond to the CORE volatile compounds. The heatmap illustrates differences in the volatile profiles between powdered/baker’s yeast and fermented/sourdough formulations across the different wheat milling co-products. WWG, whole wheat germ; DWG, defatted wheat germ; DWB, defatted wheat bran; WM, wheat middlings.

**Figure 2:**
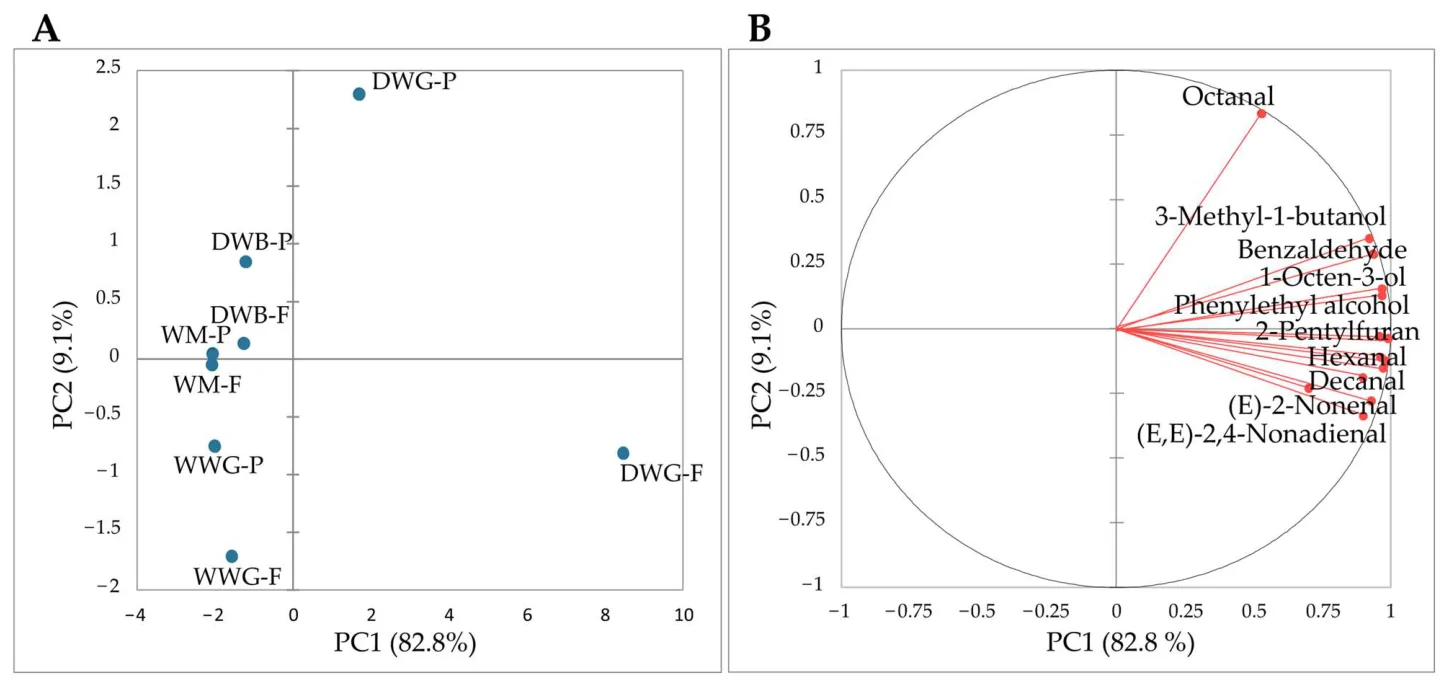
Principal component analysis (PCA) of the Z-score standardized CORE volatile organic compound (VOC) dataset for the eight *carasau* bread formulations containing wheat milling co-products, incorporated as powders in baker’s yeast breads (P-series) or after fermentation in sourdough breads (F-series). (**A**) PCA score plot. (**B**) PCA loading plot showing the contribution of the CORE VOCs to the separation of samples in the PCA space. WWG, whole wheat germ; DWG, defatted wheat germ; DWB, defatted wheat bran; WM, wheat middlings.

**Figure 3:**
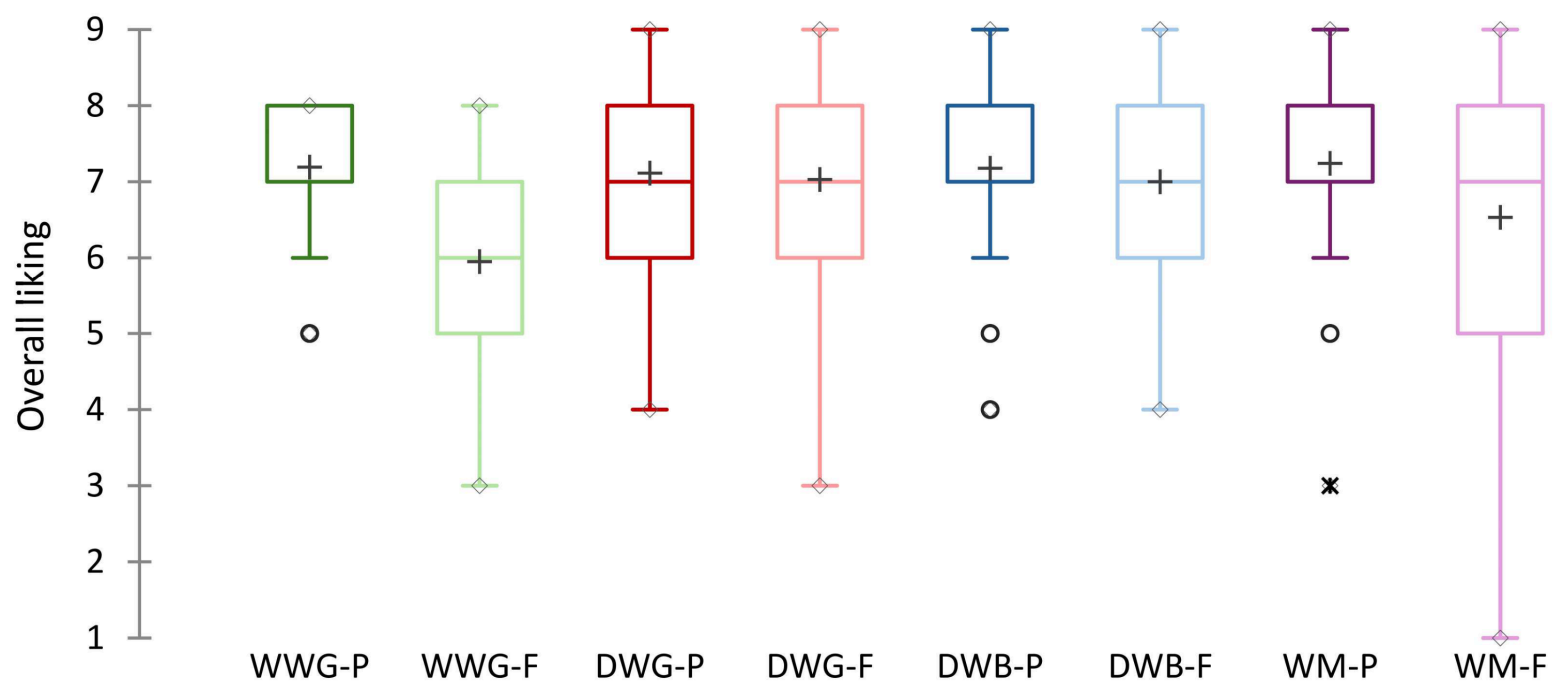
Distribution of overall liking scores for *carasau* bread formulations containing wheat milling co-products, incorporated as powders in baker’s yeast breads (P-series) or after fermentation in sourdough breads (F-series). The y-axis reports overall liking scores measured on a 9-point hedonic scale. Black crosses indicate mean values, whereas circles and asterisks denote outliers and extreme observations, respectively. WWG, whole wheat germ; DWG, defatted wheat germ; DWB, defatted wheat bran; WM, wheat middlings.

**Figure 4:**
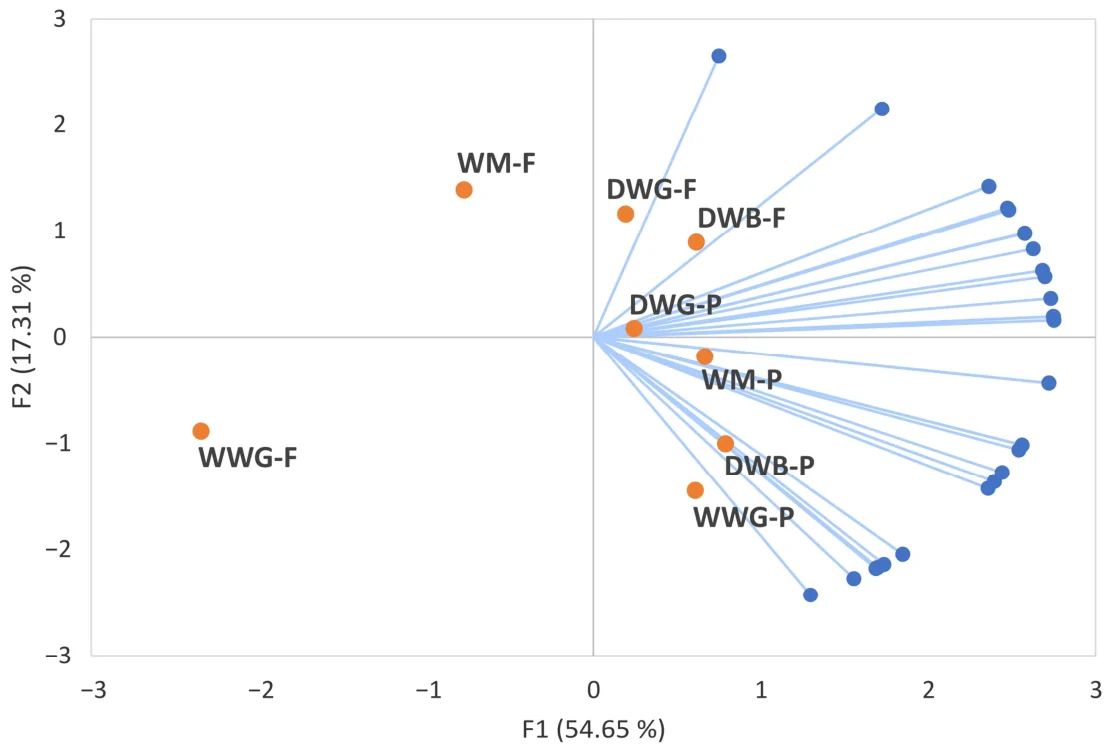
Internal preference mapping of *carasau* bread samples formulated with wheat milling co-products, incorporated as powders in baker’s yeast breads (P-series) or after fermentation in sourdough breads (F-series). The biplot was obtained from individual overall liking scores. Orange points represent bread formulations, whereas blue vectors represent individual consumer preference directions. WWG, whole wheat germ; DWG, defatted wheat germ; DWB, defatted wheat bran; WM, wheat middlings.

**Figure 5:**
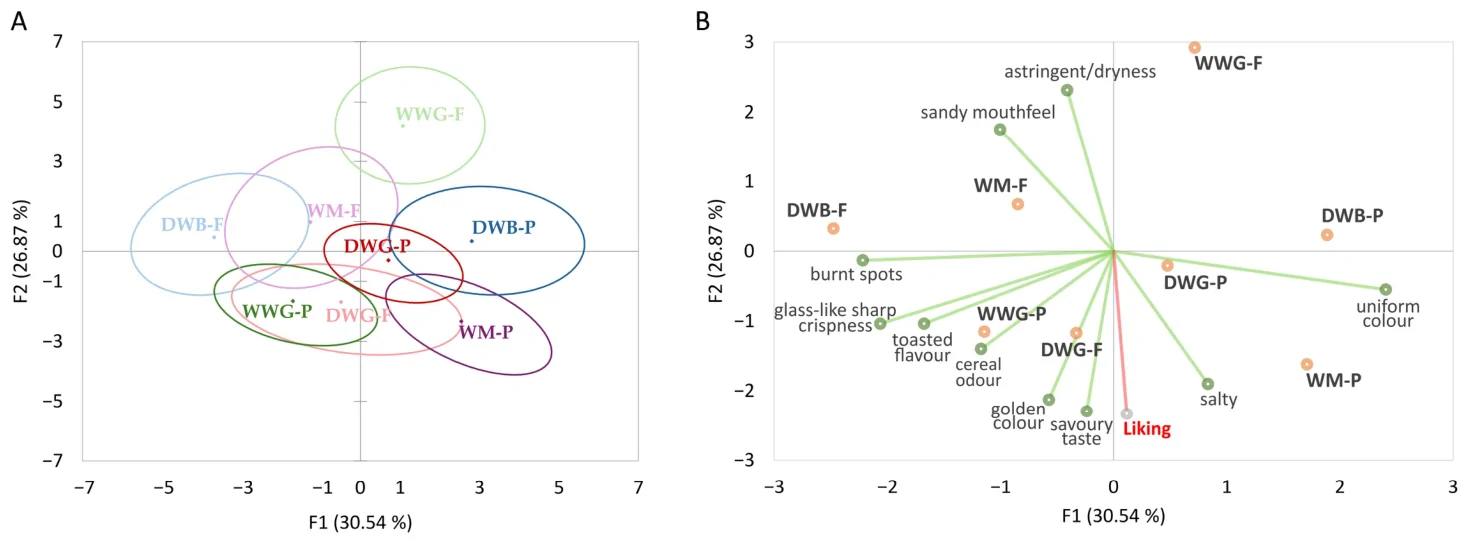
Correspondence analysis of CATA citation frequencies for the eight *carasau* bread formulations containing wheat milling co-products, incorporated as powders in baker’s yeast breads (P-series) or after fermentation in sourdough breads (F-series). (**A**) Bootstrap confidence ellipses representing the uncertainty associated with the estimated position of each formulation. (**B**) Biplot showing the associations between *carasau* bread formulations and sensory descriptors, with overall liking projected as a supplementary variable. WWG, whole wheat germ; DWG, defatted wheat germ; DWB, defatted wheat bran; WM, wheat middlings.

**Figure 6:**
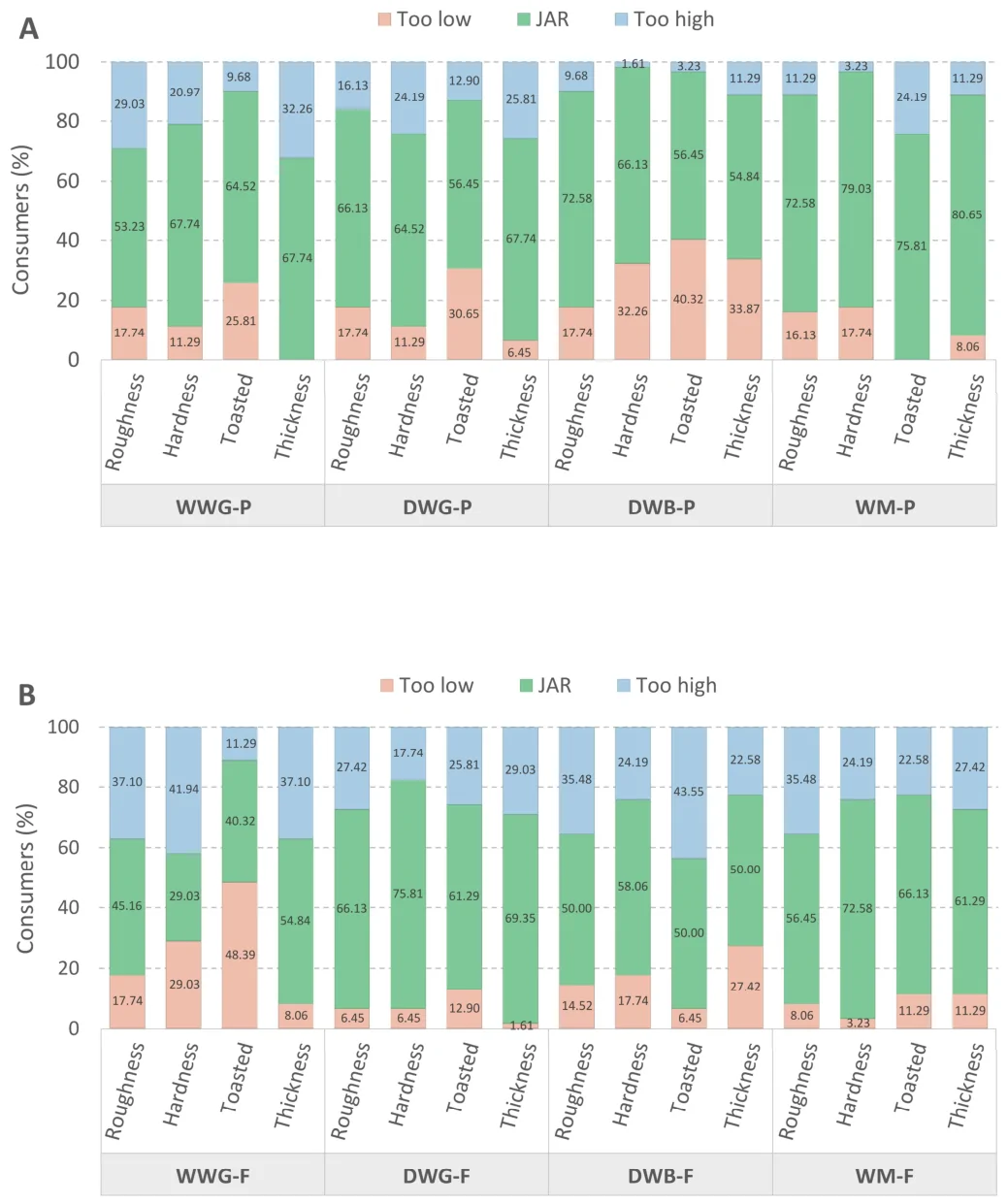
JAR profiles of *carasau* bread formulations containing wheat milling co-products, incorporated as powders in baker’s yeast breads (P-series) or after fermentation in sourdough breads (F-series). Stacked bars show the percentages of consumers rating roughness, hardness, toasted intensity, and thickness as too low, just-about-right (JAR), or too high. (**A**) Bread samples containing powdered co-product. (**B**) Bread samples containing fermented co-product. WWG, whole wheat germ; DWG, defatted wheat germ; DWB, defatted wheat bran; WM, wheat middlings.

**Figure 7:**
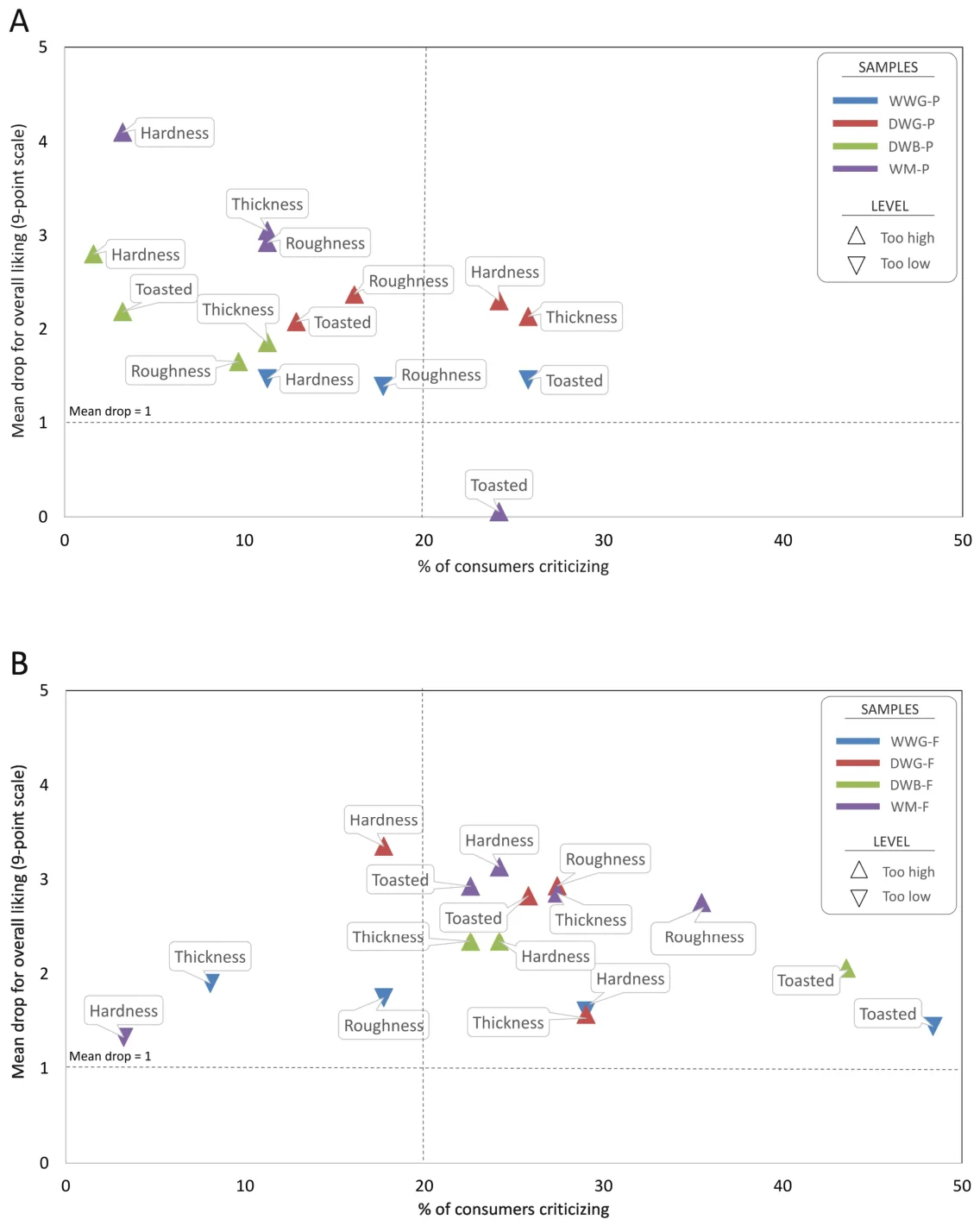
Penalty chart for *carasau* bread containing wheat milling co-products, incorporated as powders in baker’s yeast breads (P-series; **A**) or after fermentation in sourdough breads (F-series; **B**). The x-axis shows the percentage of consumers selecting each non-JAR response, whereas the y-axis reports the corresponding mean drop in overall liking. The vertical reference line indicates the thresholds of 20% of consumers, while the horizontal line indicates that a mean drop greater than 1 point on the nine-point liking scale was used as the hedonic relevance criterion. Each point represents a formulation–attribute–deviation combination. Points located in the upper-right quadrant identify deviations considered relevant for product optimization. WWG, whole wheat germ; DWG, defatted wheat germ; DWB, defatted wheat bran; WM, wheat middlings.

**Table 1:** Dough formulations used for *carasau* bread formulations containing wheat milling co-products, incorporated as powders in baker’s yeast breads (P-series) or after fermentation in sourdough breads (F-series).

Formulation	Semolina (kg)	Tap Water ^a^ (kg)	Sourdough (kg)	Co-Product (kg)	Baker’s Yeast (kg)	Sea Salt (kg)
WWG-P	100	49.00	-	5.60	1.50	1.80
WWG-F	100	33.70	23.00	-	0.13	1.80
DWG-P	100	51.00	-	5.70	1.50	1.80
DWG-F	100	40.45	20.00	-	0.13	1.80
DWB-P	100	50.60	-	5.75	1.50	1.80
DWB-F	100	38.05	23.00	-	0.13	1.80
WM-P	100	51.50	-	5.60	1.50	1.80
WM-F	100	35.85	25.00	-	0.13	1.80

WWG, whole wheat germ; DWG, defatted wheat germ; DWB, defatted wheat bran; WM, wheat middlings. ^a^ Tap water in F-doughs was adjusted to account for the water contained in the sourdough.

**Table 2:** Physicochemical properties of *carasau* bread formulations containing wheat milling co-products, incorporated as powders in baker’s yeast breads (P-series) or after fermentation in sourdough breads (F-series).

	Moisture	Ash	pH	TTA	Lactic Acid	Acetic Acid	Colour		
	(%)	(% d.w.)		mL 0.1 N NaOH	(g/kg d.w.)	(g/kg d.w.)	L *	a *	b *
WWG-P	6.76 ± 0.03 ^b^	2.98 ± 0.01 ^b^	6.22 ± 0.05 ^ab^	5.10 ± 0.23 ^e^	n.d.	0.29 ± 0.02 ^a^	70.94 ± 1.13 ^e^	4.54 ± 1.00 ^ab^	30.00 ± 1.66 ^e^
WWG-F	7.06 ± 0.12 ^a^	2.94 ± 0.02 ^b^	5.86 ± 0.04 ^c^	6.50 ± 0.12 ^d^	1.74 ± 0.02 ^d^	0.22 ± 0.01 ^b^	71.51 ± 1.80 ^de^	3.83 ± 1.19 ^bc^	27.62 ± 2.33 ^f^
DWG-P	7.01 ± 0.06 ^ab^	3.03 ± 0.01 ^a^	6.17 ± 0.01 ^b^	4.83 ± 0.08 ^e^	n.d.	0.22 ± 0.01 ^b^	75.20 ± 1.04 ^a^	4.33 ± 0.59 ^ab^	34.86 ± 1.20 ^a^
DWG-F	5.61 ± 0.05 ^e^	2.99 ± 0.04 ^ab^	5.38 ± 0.03 ^d^	9.01 ± 0.01 ^b^	4.93 ± 0.17 ^b^	0.14 ± 0.00 ^c^	75.00 ± 1.22 ^ab^	4.82 ± 0.59 ^a^	33.51 ± 1.45 ^b^
DWB-P	6.47 ± 0.13 ^c^	2.93 ± 0.09 ^abc^	6.20 ± 0.06 ^ab^	4.12 ± 0.11 f	n.d.	0.24 ± 0.01 ^b^	75.39 ± 1.27 ^a^	4.05 ± 0.48 ^bc^	31.46 ± 1.14 ^cd^
DWB-F	6.10 ± 0.08 ^d^	2.82 ± 0.04 ^c^	5.23 ± 0.04 ^e^	8.33 ± 0.28 ^c^	4.53 ± 0.01 ^c^	0.28 ± 0.01 ^a^	74.51 ± 1.15 ^abc^	4.89 ± 0.54 ^a^	31.17 ± 0.98 ^de^
WM-P	5.68 ± 0.15 ^e^	3.02 ± 0.01 ^a^	6.30 ± 0.03 ^a^	4.78 ± 0.06 ^e^	n.d.	0.27 ± 0.03 ^ab^	73.98 ± 1.16 ^bc^	3.71 ± 0.52 ^c^	32.60 ± 1.28 ^bc^
WM-F	5.56 ± 0.06 ^e^	2.99 ± 0.01 ^b^	4.97 ± 0.02 ^f^	11.37 ± 0.33 ^a^	6.14 ± 0.41 ^a^	0.17 ± 0.02 ^c^	73.11 ± 1.99 ^cd^	3.60 ± 0.99 ^bc^	30.21 ± 2.16 ^de^

Values are expressed as mean ± standard deviation. Different letters in the column indicate significant differences (*p* ≤ 0.05). d.w. refers to dry weight. n.d., not detected. WWG, whole wheat germ; DWG, defatted wheat germ; DWB, defatted wheat bran; WM, wheat middlings.

**Table 3:** Texture parameters of *carasau* bread formulations containing wheat milling co-products, incorporated as powders in baker’s yeast breads (P-series) or after fermentation in sourdough breads (F-series).

	First Break Force	Maximum Break Force	Work of Deformation	Number of Peaks	Gradient
	(N)	(N)			(N/s)
WWG-P	3.246 ± 0.938 ^ab^	5.730 ± 1.357 ^a^	2270.8 ± 514.6 ^a^	12.15 ± 3.47 ^ns^	1.169 ± 0.489 ^a^
WWG-F	3.382 ± 1.066 ^ab^	5.512 ± 1.496 ^ab^	1987.4 ± 597.0 ^ab^	11.30 ± 3.95 ^ns^	0.980 ± 0.458 ^ab^
DWG-P	3.170 ± 1.321 ^ab^	4.880 ± 1.145 ^ab^	1890.3 ± 763.2 ^abc^	11.10 ± 3.86 ^ns^	0.940 ± 0.730 ^ab^
DWG-F	3.281 ± 1.034 ^ab^	4.696 ± 0.876 ^ab^	1316.0 ± 398.4 ^c^	9.44 ± 2.48 ^ns^	0.974 ± 0.493 ^ab^
DWB-P	3.619 ± 1.206 ^a^	4.365 ± 1.055 ^b^	1880.2 ± 719.2 ^abc^	9.78 ± 3.12 ^ns^	0.824 ± 0.632 ^abc^
DWB-F	3.292 ± 0.858 ^ab^	5.026 ± 1.270 ^ab^	1684.6 ± 531.4 ^bc^	10.34 ± 3.62 ^ns^	0.747 ± 0.399 ^bc^
WM-P	2.829 ± 0.622 ^ab^	4.496 ± 0.842 ^b^	2061.4 ± 389.9 ^ab^	11.16 ± 2.29 ^ns^	0.674 ± 0.169 ^cd^
WM-F	2.623 ± 0.505 ^b^	4.544 ± 1.209 ^b^	2081.1 ± 829.1 ^ab^	11.45 ± 3.62 ^ns^	0.481 ± 0.112 ^d^

Values are expressed as mean ± standard deviation. Different letters in the column indicate significant differences (*p* ≤ 0.05). ns = not significant. WWG, whole wheat germ; DWG, defatted wheat germ; DWB, defatted wheat bran; WM, wheat middlings.

**Table 4:** Fold changes in CORE volatile organic compounds (VOCs) between *carasau* bread formulations containing wheat milling co-products, incorporated as powders in baker’s yeast breads (P-series) or after fermentation in sourdough breads (F-series).

VOC	WWG	DWG	DWB	WM
3-Methyl-1-butanol	0.75 ↓	1.22 ↑	1.04 ≈	0.77 ↓
Hexanal	1.13 ≈	4.38 ↑↑	0.88 ≈	1.39 ↑
1-Hexanol	0.91 ≈	1.72 ↑	1.48 ↑	1.14 ≈
Heptanal	1.03 ≈	2.44 ↑↑	0.68 ↓	0.93 ≈
(Z)-2-Heptenal	1.11 ≈	12.91 ↑↑↑	1.59 ↑	2.20 ↑↑
Benzaldehyde	1.37 ↑	1.55 ↑	1.16 ≈	0.95 ≈
1-Octen-3-ol	1.19 ≈	2.32 ↑↑	0.73 ↓	0.92 ≈
2-Pentylfuran	1.43 ↑	4.12 ↑↑	1.90 ↑	3.20 ↑↑
Octanal	0.79 ↓	0.81 ≈	0.86 ≈	1.09 ≈
Nonanal	1.08 ≈	1.31 ↑	0.68 ↓	0.67 ↓
Phenylethyl alcohol	0.49 ↓	3.90 ↑↑	2.07 ↑↑	1.07 ≈
(E)-2-Nonenal	1.55 ↑	1.18 ≈	0.92 ≈	0.99 ≈
Decanal	1.28 ↑	1.53 ↑	0.92 ≈	0.71 ↓
(E,E)-2,4-Nonadienal	1.16 ≈	4.55 ↑↑	3.21 ↑↑	1.37 ↑

Symbols (↓, ≈, ↑, ↑↑, ↑↑↑) indicate the magnitude and direction of the change in the fermented/sourdough formulation relative to the corresponding powdered/baker’s yeast formulation: ↓, FC < 0.80; ≈, 0.80 ≤ FC ≤ 1.20; ↑, 1.20 < FC ≤ 2.00; ↑↑, 2.00 < FC ≤ 5.00; ↑↑↑, FC > 5.00. WWG, whole wheat germ; DWG, defatted wheat germ; DWB, defatted wheat bran; WM, wheat middlings.

**Table 5:** CATA citation frequencies for *carasau* bread formulations containing wheat milling co-products, incorporated as powders in baker’s yeast breads (P-series) or after fermentation in sourdough breads (F-series). Values are expressed as proportions of consumers selecting each descriptor. Differences among formulations were evaluated using Cochran’s Q test followed, when significant, by pairwise McNemar tests with Bonferroni correction.

Descriptors	WWG-P	WWG-F	DWG-P	DWG-F	DWB-P	DWB-F	WM-P	WM-F
Burnt spots	0.565 ^bc^	0.468 ^abc^	0.355 ^a^	0.597 ^cd^	0.387 ^ab^	0.774 ^d^	0.452 ^abc^	0.565 ^bc^
Golden colour	0.855 ^b^	0.645 ^a^	0.887 ^b^	0.839 ^b^	0.710 ^ab^	0.774 ^ab^	0.839 ^b^	0.806 ^ab^
Uniform colour	0.435 ^ab^	0.613 ^bcd^	0.710 ^def^	0.629 ^cde^	0.806 ^ef^	0.371 ^a^	0.887 ^f^	0.516 ^abc^
Semolina odour	0.468 ^ab^	0.500 ^abc^	0.645 ^bc^	0.565 ^abc^	0.548 ^abc^	0.661 ^c^	0.403 ^a^	0.597 ^bc^
Cereal odour	0.613 ^b^	0.435 ^ab^	0.419 ^a^	0.532 ^ab^	0.500 ^ab^	0.581 ^ab^	0.516 ^ab^	0.435 ^ab^
Toasted flavour	0.855 ^c^	0.565 ^a^	0.758 ^bc^	0.839 ^c^	0.597 ^ab^	0.935 ^c^	0.597 ^ab^	0.645 ^ab^
Wholegrain flavour	0.677 ^d^	0.419 ^bc^	0.290 ^abc^	0.306 ^abc^	0.306 ^abc^	0.452 ^c^	0.194 ^a^	0.242 ^ab^
Savoury taste	0.855 ^c^	0.387 ^a^	0.726 ^bc^	0.806 ^c^	0.710 ^bc^	0.613 ^b^	0.677 ^bc^	0.597 ^b^
Bland taste	0.290 ^ab^	0.323 ^b^	0.306 ^b^	0.226 ^ab^	0.274 ^ab^	0.258 ^ab^	0.113 ^a^	0.242 ^ab^
Bitter taste	0.000 ^a^	0.032 ^ab^	0.016 ^ab^	0.177 ^abc^	0.081 ^ab^	0.323 ^c^	0.113 ^ab^	0.194 ^bc^
Slightly acidic	0.210 ^a^	0.145 ^a^	0.258 ^a^	0.452 ^b^	0.097 ^a^	0.516 ^b^	0.113 ^a^	0.758 ^c^
Fragile/easily breakable	0.468 ^ab^	0.484 ^ab^	0.484 ^ab^	0.484 ^ab^	0.806 ^c^	0.548 ^b^	0.452 ^ab^	0.323 ^a^
Glass-like/sharp crispness	0.645 ^d^	0.323 ^ab^	0.484 ^bcd^	0.387 ^bc^	0.177 ^a^	0.532 ^cd^	0.484 ^bcd^	0.516 ^cd^
Sandy mouthfeel	0.242 ^abc^	0.290 ^bc^	0.290 ^bc^	0.145 ^ab^	0.242 ^abc^	0.274 ^bc^	0.081 ^a^	0.355 ^c^
Astringent/dryness	0.290 ^ab^	0.677 ^c^	0.210 ^a^	0.274 ^ab^	0.226 ^ab^	0.323 ^ab^	0.161 ^a^	0.403 ^b^
Stale flavour	0.0 ^a^	0.242 ^b^	0.0 ^a^	0.081 ^a^	0.0 ^a^	0.0 ^a^	0.0 ^a^	0.016 ^a^

Different letters within rows indicate significant differences among samples (*p* < 0.05). WWG, whole wheat germ; DWG, defatted wheat germ; DWB, defatted wheat bran; WM, wheat middlings.

## Data Availability

The original contributions presented in the study are included in the article/Supplementary Materials, further inquiries can be directed to the corresponding authors.

## Supplementary Materials

The following supporting information can be downloaded at: https://www.mdpi.com/article/10.3390/foods15203754/s1, Figure S1: Exploratory hierarchical cluster analysis; Figure S2: Total Penalty Scores (TPS) for JAR attribute deviations; Table S1: Properties of doughs and sourdoughs; Table S2: Experimental linear retention indices (LRIs) of volatile organic compounds; Table S3: Normalized relative abundance of target volatile organic compounds (VOCs); Table S4: Direction-specific JAR penalty analysis.

## References

[1] Sarfaraz A., Azizi M.H., Ahmadi Gavlighi H., Barzegar M. (2017). Physicochemical and Functional Characterization of Wheat Milling Co-Products: Fine Grinding to Achieve High Fiber Antioxidant-Rich Fractions. J. Cereal Sci..

[2] Suanno C., Marincich L., Corneti S., Aloisi I., Pincigher L., Papi E., Parrotta L., Antognoni F., Del Duca S. (2025). Biochemical Analysis of Wheat Milling By-Products for Their Valorization as Potential Food Ingredients. Int. J. Mol. Sci..

[3] Ferreira D.M., Coscueta E.R., Brassesco M.E., Pintado M. (2026). Processing and Valorization of Wheat Bran, Germ and Their Fractions: An Evidence-Graded Review of Composition, Technologies and Applications. Foods.

[4] Verni M., Rizzello C.G., Coda R. (2019). Fermentation Biotechnology Applied to Cereal Industry By-Products: Nutritional and Functional Insights. Front. Nutr..

[5] Perri G., Greco Miani M., Amendolagine G., Pontonio E., Rizzello C.G. (2022). Defatted Durum Wheat Germ to Produce Type-II and III Sourdoughs: Characterization and Use as Bread Ingredient. LWT.

[6] Heiniö R.L., Noort M.W.J., Katina K., Alam S.A., Sozer N., De Kock H.L., Hersleth M., Poutanen K. (2016). Sensory Characteristics of Wholegrain and Bran-Rich Cereal Foods—A Review. Trends Food Sci. Technol..

[7] Pontonio E., Lorusso A., Gobbetti M., Rizzello C.G. (2017). Use of Fermented Milling By-Products as Functional Ingredient to Develop a Low-Glycaemic Index Bread. J. Cereal Sci..

[8] Bartkiene E., Jomantaite I., Mockus E., Ruibys R., Baltusnikiene A., Santini A., Zokaityte E. (2021). The Contribution of Extruded and Fermented Wheat Bran to the Quality Parameters of Wheat Bread, Including the Profile of Volatile Compounds and Their Relationship with Emotions Induced for Consumers. Foods.

[9] Rizzello C.G., Coda R., Mazzacane F., Minervini D., Gobbetti M. (2012). Micronized By-Products from Debranned Durum Wheat and Sourdough Fermentation Enhanced the Nutritional, Textural and Sensory Features of Bread. Food Res. Int..

[10] Catzeddu P., Fois S., Tolu V., Sanna M., Braca A., Vitangeli I., Anedda R., Roggio T. (2023). Quality Evaluation of Fresh Pasta Fortified with Sourdough Containing Wheat Germ and Wholemeal Semolina. Foods.

[11] Rizzello C.G., Nionelli L., Coda R., Di Cagno R., Gobbetti M. (2010). Use of Sourdough Fermented Wheat Germ for Enhancing the Nutritional, Texture and Sensory Characteristics of the White Bread. Eur. Food Res. Technol..

[12] Sanna M., Fois S., Falchi G., Campus M., Roggio T., Catzeddu P. (2019). Effect of Liquid Sourdough Technology on the Pre-Biotic, Texture, and Sensory Properties of a Crispy Flatbread. Food Sci. Biotechnol..

[13] Meyners M., Castura J.C., Carr B.T. (2013). Existing and New Approaches for the Analysis of CATA Data. Food Qual. Prefer..

[14] Aguiar L.A.D., Rodrigues D.B., Queiroz V.A.V., Melo L., Pineli L.d.L.d.O. (2020). Comparison of Two Rapid Descriptive Sensory Techniques for Profiling and Screening of Drivers of Liking of Sorghum Breads. Food Res. Int..

[15] Rødbotten M., Tomic O., Holtekjølen A.K., Grini I.S., Lea P., Granli B.S., Grimsby S., Sahlstrøm S. (2015). Barley Bread with Normal and Low Content of Salt; Sensory Profile and Consumer Preference in Five European Countries. J. Cereal Sci..

[16] Sanna M., Sanna S., Serra M., Roggio T., Catzeddu P., Sanna V. (2026). From Composition to Acceptance: Linking Nutritional, Structural and Sensory Attributes in Clean-Label Breads. Foods.

[17] Stone H., Bleibaum R.N., Thomas H.A. (2012). Sensory Evaluation Practices.

[18] Challacombe C.A., Seetharaman K., Duizer L.M. (2011). Sensory Characteristics and Consumer Acceptance of Bread and Cracker Products Made from Red or White Wheat. J. Food Sci..

[19] Narayanan P., Chinnasamy B., Jin L., Clark S. (2014). Use of Just-about-Right Scales and Penalty Analysis to Determine Appropriate Concentrations of Stevia Sweeteners for Vanilla Yogurt. J. Dairy Sci..

[20] Rothman L., Parker M.J. (2009). Just-About-Right (JAR) Scales: Design, Usage, Benefits, and Risks.

[21] Ortega-Heras M., Gómez I., De Pablos-Alcalde S., González-Sanjosé M.L. (2019). Application of the Just-About-Right Scales in the Development of New Healthy Whole-Wheat Muffins by the Addition of a Product Obtained from White and Red Grape Pomace. Foods.

[22] Drakula S., Mustač N.Č., Novotni D., Voučko B., Krpan M., Hruškar M., Ćurić D. (2022). Optimization and Validation of a HS-SPME/GC–MS Method for the Analysis of Gluten-Free Bread Volatile Flavor Compounds. Food Anal. Methods.

[23] Pétel C., Onno B., Prost C. (2017). Sourdough Volatile Compounds and Their Contribution to Bread: A Review. Trends Food Sci. Technol..

[24] Heenan S.P., Dufour J.-P., Hamid N., Harvey W., Delahunty C.M. (2009). Characterisation of Fresh Bread Flavour: Relationships between Sensory Characteristics and Volatile Composition. Food Chem..

[25] Warburton A., Silcock P., Eyres G.T. (2022). Impact of Sourdough Culture on the Volatile Compounds in Wholemeal Sourdough Bread. Food Res. Int..

[26] Sanna V., Tolu V., Piu P.P., Sanna M., Piras D., Fois S., Braca A., Roggio T., Catzeddu P. (2026). Unlocking Antioxidant Potential in Wheat By-Products Through Microbial Bioprocessing: An Integrated Extraction–QUENCHER Assessment. Molecules.

[27] Fois S., Tolu V., Sanna V., Loddo A., Sanna M., Piu P.P., Piras D., Roggio T., Catzeddu P. (2025). Valorizing Carasau Bread Residue Through Sourdough Fermentation: From Bread Waste to Bread Taste. Microorganisms.

[28] Plessas S., Bekatorou A., Gallanagh J., Nigam P., Koutinas A.A., Psarianos C. (2008). Evolution of Aroma Volatiles during Storage of Sourdough Breads Made by Mixed Cultures of *Kluyveromyces marxianus* and *Lactobacillus delbrueckii* ssp. *bulgaricus* or *Lactobacillus helveticus*. Food Chem..

[29] Stein S.E. (1999). An Integrated Method for Spectrum Extraction and Compound Identification from Gas Chromatography/Mass Spectrometry Data. J. Am. Soc. Mass Spectrom..

[30] Van Den Dool H., Kratz P.D. (1963). A Generalization of the Retention Index System Including Linear Temperature Programmed Gas—Liquid Partition Chromatography. J. Chromatogr. A.

[31] Ares G., Tárrega A., Izquierdo L., Jaeger S.R. (2014). Investigation of the Number of Consumers Necessary to Obtain Stable Sample and Descriptor Configurations from Check-All-That-Apply (CATA) Questions. Food Qual. Prefer..

[32] (2014). Sensory Analysis-Methodology-General Guidance for Conducting Hedonic Tests with Consumers in a Controlled Area.

[33] (2017). Sensory Analysis-Methodology-General Guidance.

[34] Lucak C.L., Delwiche J.F. (2009). Efficacy of Various Palate Cleansers with Representative Foods. Chemosens. Percept..

[35] Peryam D.R., Pilgrim F.J. (1957). Hedonic Scale Method of Measuring Food Preferences. Food Technol..

[36] Lawless H.T., Heymann H. (2010). Sensory Evaluation of Food: Principles and Practices.

[37] Rodrigues I., Magalhaes D.R., Trindade M.A. (2022). Use of Focus Group as Selection Method of Descriptors for Check-All-That-Apply (CATA) for Sensory Characteristics of Hot Dogs. Foods.

[38] Ares G., Jaeger S.R., Bava C.M., Chheang S.L., Jin D., Gimenez A., Vidal L., Fiszman S.M., Varela P. (2013). CATA Questions for Sensory Product Characterization: Raising Awareness of Biases. Food Qual. Prefer..

[39] Jaeger S.R., Ares G. (2014). Lack of Evidence That Concurrent Sensory Product Characterisation Using CATA Questions Bias Hedonic Scores. Food Qual. Prefer..

[40] Jaeger S.R., Giacalone D., Roigard C.M., Pineau B., Vidal L., Giménez A., Frøst M.B., Ares G. (2013). Investigation of Bias of Hedonic Scores When Co-Eliciting Product Attribute Information Using CATA Questions. Food Qual. Prefer..

[41] Geffroy O., Maza E., Lytra G., Chervin C. (2024). ‘Liking Then CATA’ or ‘CATA Then Liking’? Impact of the Hedonic Question Positioning on the Wine Sensory Description and Appreciation. OENO One.

[42] Ares G., Jaeger S.R. (2013). Check-All-That-Apply Questions: Influence of Attribute Order on Sensory Product Characterization. Food Qual. Prefer..

[43] Ares G., Varela P., Ares G., Varela P. (2018). Consumer-Based Methodologies for Sensory Characterization. Methods in Consumer Research, Volume 1.

[44] Li B., Hayes J.E., Ziegler G.R. (2014). Just-about-Right and Ideal Scaling Provide Similar Insights into the Influence of Sensory Attributes on Liking. Food Qual. Prefer..

[45] JASP Team 2026. JASP (Version 0.98.1): [Computer Software]. https://jasp-stats.org.

[46] García-Gómez B., Fernández-Canto N., Vázquez-Odériz M.L., Quiroga-García M., Muñoz-Ferreiro N., Romero-Rodríguez M.Á. (2022). Sensory Descriptive Analysis and Hedonic Consumer Test for Galician Type Breads. Food Control.

[47] Wagner A., Irmler J., Nagypál J., Bach P., Ludwig M., Will F., Schweiggert R., Steingass C.B. (2023). HS–SPME–GC–MS Profiling and Sensory Analyses of Juices from Red-Fleshed ‘Weirouge’ Apples Made with Innovative and Conventional Dejuicing Systems. Eur. Food Res. Technol..

[48] Popper R., Varela L., Ares G. (2014). Use of Just-About-Right Scales in Consumer Research. Novel Techniques in Sensory Characterization and Consumer Profiling.

[49] Fois S., Sanna M., Stara G., Roggio T., Catzeddu P. (2011). Rheological Properties and Baking Quality of Commercial Durum Wheat Meals Used to Make Flat Crispy Bread. Eur. Food Res. Technol..

[50] De Bondt Y., Verdonck C., Brandt M.J., De Vuyst L., Gänzle M.G., Gobbetti M., Zannini E., Courtin C.M. (2024). Wheat Sourdough Breadmaking: A Scoping Review. Annu. Rev. Food Sci. Technol..

[51] Rizzello C.G., Nionelli L., Coda R., De Angelis M., Gobbetti M. (2010). Effect of Sourdough Fermentation on Stabilisation, and Chemical and Nutritional Characteristics of Wheat Germ. Food Chem..

[52] Longo A., Amendolagine G., Miani M.G., Rizzello C.G., Verni M. (2024). Effect of Air Classification and Enzymatic and Microbial Bioprocessing on Defatted Durum Wheat Germ: Characterization and Use as Bread Ingredient. Foods.

[53] Bakke A., Vickers Z. (2007). Consumer Liking of Refined and Whole Wheat Breads. J. Food Sci..

[54] Bakke A., Vickers Z. (2011). Effects of Bitterness, Roughness, PROP Taster Status, and Fungiform Papillae Density on Bread Acceptance. Food Qual. Prefer..

[55] Bin Q., Peterson D.G. (2016). Identification of Bitter Compounds in Whole Wheat Bread Crumb. Food Chem..

[56] Jiang D., Peterson D.G. (2013). Identification of Bitter Compounds in Whole Wheat Bread. Food Chem..

[57] Plaehn D., Horne J. (2008). A Regression-Based Approach for Testing Significance of “Just-about-Right” Variable Penalties. Food Qual. Prefer..

[58] Paries M., Bougeard S., Vigneau E. (2022). Multivariate Analysis of Just-About-Right Data with Optimal Scaling Approach. Food Qual. Prefer..

[59] Rinaldi M., Paciulli M., Caligiani A., Sgarbi E., Cirlini M., Dall’Asta C., Chiavaro E. (2015). Durum and Soft Wheat Flours in Sourdough and Straight-Dough Bread-Making. J. Food Sci. Technol..

[60] Codină G.G., Sarion C., Dabija A. (2021). Effects of Dry Sourdough on Bread-Making Quality and Acrylamide Content. Agronomy.

[61] Antognoni F., Mandrioli R., Potente G., Taneyo Saa D.L., Gianotti A. (2019). Changes in Carotenoids, Phenolic Acids and Antioxidant Capacity in Bread Wheat Doughs Fermented with Different Lactic Acid Bacteria Strains. Food Chem..

[62] Purlis E. (2010). Browning Development in Bakery Products—A Review. J. Food Eng..

[63] Arora K., Ameur H., Polo A., Di Cagno R., Rizzello C.G., Gobbetti M. (2021). Thirty Years of Knowledge on Sourdough Fermentation: A Systematic Review. Trends Food Sci. Technol..

[64] Bahal G., Sudha M.L., Ramasarma P.R. (2013). Wheat Germ Lipoxygenase: Its Effect on Dough Rheology, Microstructure, and Bread Making Quality. Int. J. Food Prop..

[65] Grebenteuch S., Kroh L.W., Drusch S., Rohn S. (2021). Formation of Secondary and Tertiary Volatile Compounds Resulting from the Lipid Oxidation of Rapeseed Oil. Foods.

[66] Ares G., Jaeger S.R., Delarue J., Lawlor J.B., Rogeaux M. (2015). Check-All-That-Apply (CATA) Questions with Consumers in Practice: Experimental Considerations and Impact on Outcome. Rapid Sensory Profiling Techniques.

[67] Vigneau E., Cariou V., Giacalone D., Berget I., Llobell F. (2022). Combining Hedonic Information and CATA Description for Consumer Segmentation. Food Qual. Prefer..

