# Supplementary material for: Valorization of Wheat Milling Co-Products in Carasau Bread: Effects of Their Addition as Powders or After Fermentation on Physicochemical Properties, Volatile Profile and Sensory Characteristics
Source: Foods. 2026 Oct 21;15(20):3754. doi: 10.3390/foods15203754 (PMC13704362; doi:10.3390/foods15203754)
Supplement: Supplementary file 1 [file foods-15-03754-s001.zip › Figure S1.pdf]

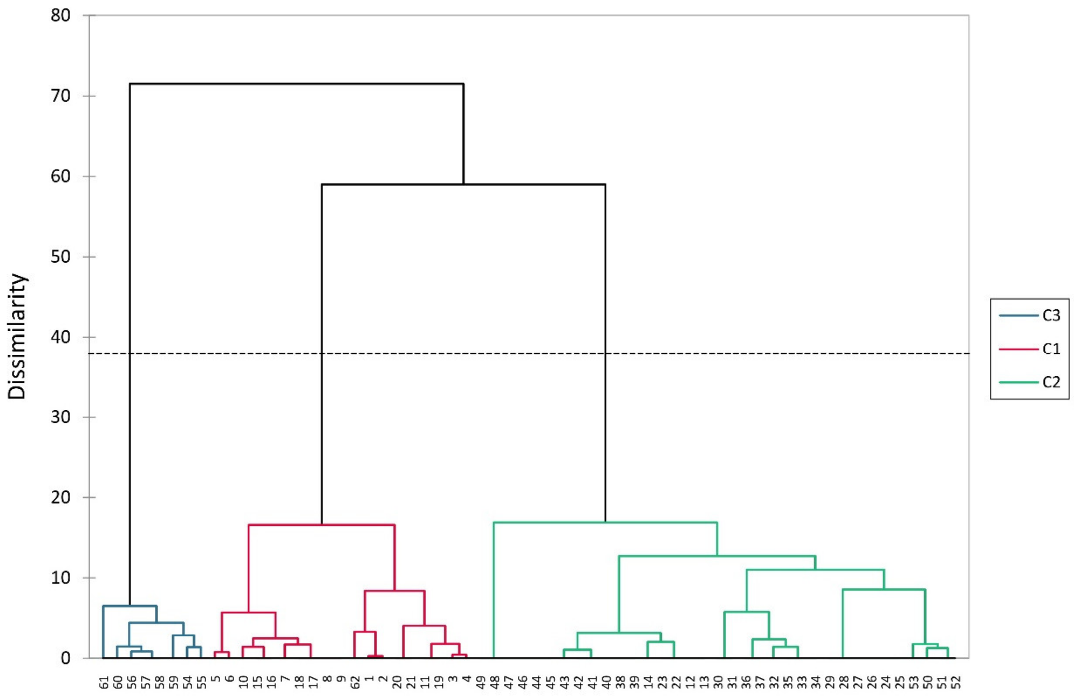

Figure S1. Exploratory hierarchical cluster analysis of the 62 consumers based on overall liking scores for the eight *carasau* bread formulations containing wheat milling co-products. Assessor scores were centered and scaled prior to clustering. Euclidean distance and Ward's agglomeration method were used. The dendrogram identified three consumer clusters: C1 (n = 19), C2 (n = 35), and C3 (n = 8). Based on the original, non-standardized liking scores, C1 showed comparatively lower overall liking, with greater appreciation of powdered formulations, particularly WWG-P and DWB-P. C2 exhibited generally favorable liking scores, especially for DWB-F, DWB-P, and DWG-F. C3 showed consistently high liking scores, particularly for DWG-P, DWG-F, WM-P, and WM-F. The y-axis represents dissimilarity among consumers, with lower linkage levels indicating greater similarity in preference patterns. Given the relatively limited sample size, the resulting segmentation was interpreted as exploratory.
