# Supplementary material for: Valorization of Wheat Milling Co-Products in Carasau Bread: Effects of Their Addition as Powders or After Fermentation on Physicochemical Properties, Volatile Profile and Sensory Characteristics
Source: Foods. 2026 Oct 21;15(20):3754. doi: 10.3390/foods15203754 (PMC13704362; doi:10.3390/foods15203754)
Supplement: Supplementary file 1 [file foods-15-03754-s001.zip › Figure S2.pdf]

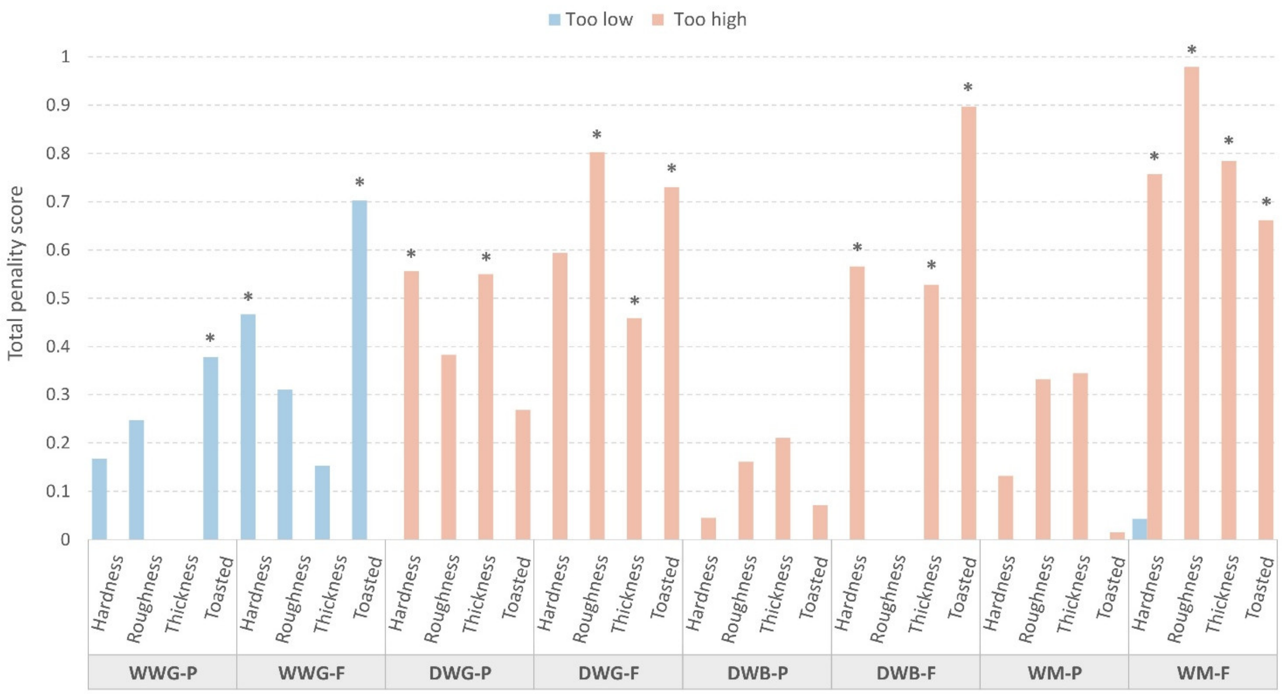

Figure S2. Total Penalty Scores (TPS) for JAR attribute deviations associated with overall liking of *carasau* formulations containing wheat milling co-products. TPS values were calculated separately for each attribute and direction of deviation ("too low" and "too high") using a weighted-penalty approach: TPS = p(non-JAR) × positive mean drop, where p(non-JAR) is the proportion of consumers selecting the corresponding non-JAR category, and mean drop is the difference between the mean overall liking of consumers rating the attribute as JAR and the mean overall liking of consumers in the corresponding non-JAR category. When the mean drop was zero or negative, TPS was set to zero. "Too low" and "too high" deviations were treated separately and were not summed. A penalty was considered relevant for interpretation when the corresponding non-JAR category included at least 20% of consumers and the mean drop exceeded 1 point on the 9-point liking scale. Mean drop was unavailable only when no consumer selected the corresponding non-JAR category. Asterisks indicate non-JAR deviations considered relevant for interpretation, defined as those selected by ≥20% of consumers and associated with a mean drop in overall liking >1 point on the 9-point hedonic scale."
