# Supplementary material for: Valorization of Wheat Milling Co-Products in Carasau Bread: Effects of Their Addition as Powders or After Fermentation on Physicochemical Properties, Volatile Profile and Sensory Characteristics
Source: Foods. 2026 Oct 21;15(20):3754. doi: 10.3390/foods15203754 (PMC13704362; doi:10.3390/foods15203754)
Supplement: Supplementary file 1 [file foods-15-03754-s001.zip › Table S1.pdf]

Table S1. Properties of doughs and sourdoughs

Formulation	Total dough weight (kg)	Water on total dough (%, w/w)	Dough moisture (%)[1]	Sourdough[2]	
				pH	TTA
WWG-P	157.70	30.94	40.63±0.06	-	-
WWG-F	158.60	31.34	39.89±0.39	4.33 ± 0.00	14.64 ± 0.12
DWG-P	160.00	31.88	41.19±0.16	-	-
DWG-F	162.35	32.83	41.12±0.03	4.92 ± 0.04	18.35 ± 0.10
DWB-P	159.65	31.69	41.65±0.07	-	-
DWB-F	162.97	33.04	41.69±0.07	4.81 ± 0.06	10.72 ± 0.31
WM-P	160.40	32.11	41.49±0.07	-	-
WM-F	162.79	33.11	40.47±0.13	4.26 ± 0.01	15.89 ± 0.35

WWG, whole wheat germ; DWG, defatted wheat germ; DWB, defatted wheat bran; WM, wheat middlings; P, powdered co-product; F, fermented co-product.

[1]Moisture was calculated on dough samples left at 130 °C until a stable weight.

[2]Sourdoughs used for F-bread formulation
