# Supplementary material for: Valorization of Wheat Milling Co-Products in Carasau Bread: Effects of Their Addition as Powders or After Fermentation on Physicochemical Properties, Volatile Profile and Sensory Characteristics
Source: Foods. 2026 Oct 21;15(20):3754. doi: 10.3390/foods15203754 (PMC13704362; doi:10.3390/foods15203754)
Supplement: Supplementary file 1 [file foods-15-03754-s001.zip › Table S2.pdf]

Table S2. Experimental linear retention indices (LRIs) of volatile organic compounds identified in carasau bread samples and comparison with NIST reference retention indices.

Compound	RT (min)	Experimental LRI	NIST reference RI[1]	ΔRI[2]
Pentanal	6.620	n.c.	699	—
3-Methyl-1-butanol	7.380	n.c.	736	—
1-Pentanol	8.162	n.c.	765	—
Hexanal	9.165	803	800	+3
1-Hexanol	11.584	870	868	+2
Heptanal	12.884	906	901	+5
(Z)-2-Heptenal	15.198	963	958	+5
Benzaldehyde	15.576	972	962	+10
1-Octen-3-ol	16.092	984	980	+4
2-Pentylfuran	16.561	996	993	+3
Octanal	17.090	1009	1003	+6
3-Octen-2-one	18.551	1045	1040	+5
(E)-2-Octenal	19.413	1066	1060	+6
(E,E)-3,5-Octadien-2-one	19.866	1077	1073	+4
Nonanal	21.250	1112	1104	+8
Phenylethyl alcohol	21.756	1125	1116	+9
(E)-2-Nonenal	23.455	1170	1162	+8
Decanal	25.160	1215	1206	+9
(E,E)-2,4-Nonadienal	25.616	1228	1216	+12
(E,E)-2,4-Decadienal	29.431	1329	1317	+12

[1] NIST reference RI values correspond to the median experimental retention indices reported for semi-standard non-polar stationary phases. [2] ΔRI = experimental LRI − NIST reference RI. n.c., not calculated because the compound eluted before the first n-alkane (C8) of the reference series.
