# Supplementary material for: Valorization of Wheat Milling Co-Products in Carasau Bread: Effects of Their Addition as Powders or After Fermentation on Physicochemical Properties, Volatile Profile and Sensory Characteristics
Source: Foods. 2026 Oct 21;15(20):3754. doi: 10.3390/foods15203754 (PMC13704362; doi:10.3390/foods15203754)
Supplement: Supplementary file 1 [file foods-15-03754-s001.zip › Table S3.pdf]

Table S3. Normalized relative abundance of target volatile organic compounds (VOCs) in carasau bread formulations.

Compound	WWG-P	WWG-F	DWG-P	DWG-F	DWB-P	DWB-F	WM-P	WM-F
Pentanal	n.d.	0.0029 ± 0.0003	n.d.	n.d.	n.d.	n.d.	n.d.	0.0025 ± 0.0002
3-Methyl-1-butanol	0.0424 ± 0.0022	0.0319 ± 0.0034	0.1505 ± 0.0083	0.1842 ± 0.0362	0.0646 ± 0.0103	0.0675 ± 0.0074	0.0392 ± 0.0021	0.0301 ± 0.0007
1-Pentanol	0.0036 ± 0.0006	0.0052 ± 0.0009	n.d.	0.0201 ± 0.0106	0.0067 ± 0.0009	0.0062 ± 0.0011	0.0027 ± 0.0003	0.0051 ± 0.0003
Hexanal	0.0629 ± 0.0036	0.0709 ± 0.0102	0.1336 ± 0.0049	0.5853 ± 0.0073	0.1184 ± 0.0052	0.1038 ± 0.0121	0.0502 ± 0.0042	0.0698 ± 0.0062
1-Hexanol	0.0187 ± 0.0016	0.0170 ± 0.0027	0.0485 ± 0.0033	0.0836 ± 0.0002	0.0249 ± 0.0031	0.0369 ± 0.0032	0.0151 ± 0.0019	0.0173 ± 0.0017
Heptanal	0.0117 ± 0.0005	0.0121 ± 0.0021	0.0240 ± 0.0007	0.0585 ± 0.0096	0.0185 ± 0.0005	0.0125 ± 0.0016	0.0127 ± 0.0011	0.0118 ± 0.0009
(Z)-2-Heptenal	0.0222 ± 0.0025	0.0247 ± 0.0022	0.0135 ± 0.0003	0.1739 ± 0.0146	0.0182 ± 0.0026	0.0291 ± 0.0053	0.0186 ± 0.0034	0.0410 ± 0.0020
Benzaldehyde	0.0356 ± 0.0044	0.0488 ± 0.0072	0.1678 ± 0.0156	0.2607 ± 0.0527	0.0438 ± 0.0025	0.0509 ± 0.0060	0.0569 ± 0.0039	0.0542 ± 0.0044
1-Octen-3-ol	0.0392 ± 0.0042	0.0464 ± 0.0011	0.0692 ± 0.0049	0.1604 ± 0.0056	0.0746 ± 0.0065	0.0545 ± 0.0069	0.0317 ± 0.0054	0.0290 ± 0.0036
2-Pentylfuran	0.0290 ± 0.0026	0.0416 ± 0.0078	0.1432 ± 0.0071	0.5903 ± 0.0029	0.0494 ± 0.0011	0.0937 ± 0.0111	0.0205 ± 0.0014	0.0656 ± 0.0061
Octanal	0.0106 ± 0.0007	0.0084 ± 0.0009	0.0200 ± 0.0024	0.0161 ± 0.0022	0.0150 ± 0.0019	0.0128 ± 0.0012	0.0126 ± 0.0007	0.0137 ± 0.0010
3-Octen-2-one	0.0086 ± 0.0003	0.0074 ± 0.0013	n.d.	0.0927 ± 0.0140	n.d.	0.0060 ± 0.0005	0.0065 ± 0.0005	0.0093 ± 0.0009
(E)-2-Octenal	0.0272 ± 0.0021	0.0339 ± 0.0038	n.d.	n.d.	0.0198 ± 0.0009	0.0322 ± 0.0029	0.0258 ± 0.0024	0.0588 ± 0.0054
(E,E)-3,5-Octadien-2-one	0.0139 ± 0.0012	0.0110 ± 0.0013	n.d.	n.d.	n.d.	n.d.	n.d.	n.d.
Nonanal	0.2460 ± 0.0136	0.2667 ± 0.0314	0.6376 ± 0.0486	0.8384 ± 0.1360	0.4141 ± 0.0238	0.2808 ± 0.0121	0.3546 ± 0.0300	0.2377 ± 0.0217
Phenylethyl alcohol	0.2181 ± 0.0126	0.1060 ± 0.0123	0.4088 ± 0.0314	1.5935 ± 0.2479	0.2258 ± 0.0219	0.4665 ± 0.0557	0.1521 ± 0.0108	0.1625 ± 0.0126
(E)-2-Nonenal	0.0863 ± 0.0031	0.1337 ± 0.0115	0.1231 ± 0.0178	0.1449 ± 0.0262	0.0660 ± 0.0055	0.0606 ± 0.0107	0.0726 ± 0.0137	0.0720 ± 0.0089
Decanal	0.0473 ± 0.0025	0.0605 ± 0.0056	0.0628 ± 0.0034	0.0958 ± 0.0152	0.0389 ± 0.0048	0.0357 ± 0.0011	0.0378 ± 0.0035	0.0269 ± 0.0029
(E,E)-2,4-Nonadienal	0.0057 ± 0.0004	0.0067 ± 0.0006	0.0077 ± 0.0004	0.0350 ± 0.0043	0.0019 ± 0.0005	0.0062 ± 0.0005	0.0065 ± 0.0004	0.0089 ± 0.0012
(E,E)-2,4-Decadienal	0.0310 ± 0.0023	0.0366 ± 0.0027	n.d.	n.d.	0.0129 ± 0.0026	0.0095 ± 0.0012	0.0228 ± 0.0024	0.0379 ± 0.0051

Values are expressed as mean normalized peak-area ratios (AreaVOC/Areainternal standard) ± SD from three technical replicates prepared from the same experimental production batch. 2-Methyl-3-heptanone was used as the internal standard. A numerical value was reported only when the compound was detected in all three technical replicates; otherwise, the result was reported as n.d. (not consistently detected in all three technical replicates). Values represent relative instrumental responses and should not be interpreted as absolute or semi-quantitative concentrations.
