# Supplementary material for: Valorization of Wheat Milling Co-Products in Carasau Bread: Effects of Their Addition as Powders or After Fermentation on Physicochemical Properties, Volatile Profile and Sensory Characteristics
Source: Foods. 2026 Oct 21;15(20):3754. doi: 10.3390/foods15203754 (PMC13704362; doi:10.3390/foods15203754)
Supplement: Supplementary file 1 [file foods-15-03754-s001.zip › Table S4.pdf]

Table S4. Direction-specific JAR penalty analysis for overall liking of *carasau* bread formulations containing wheat milling co-products in powdered and fermented forms. For each sample and sensory attribute, the proportion of consumers selecting the corresponding non-JAR category (“too low” or “too high”), the associated mean drop in overall liking, the direction-specific total penalty score (TPS), and whether the deviation met the predefined criterion for relevance are reported. TPS was calculated as *p*(non-JAR) × positive mean drop, where *p*(non-JAR) is the proportion of consumers selecting the corresponding non-JAR category. When the mean drop was zero or negative, TPS was set to zero. Deviations were considered relevant for interpretation when the corresponding non-JAR category included at least 20% of consumers and the mean drop exceeded 1 point on the 9-point liking scale. Mean drop was unavailable only when no consumer selected the corresponding non-JAR category.

Sample	Attribute	Direction	Consumers (%)	Mean drop	TPS
WWG-P	Roughness	Too low	17.74%	1.394	0.247
WWG-P	Roughness	Too high	29.03%	-0.788	0.000
WWG-P	Hardness	Too low	11.29%	1.476	0.167
WWG-P	Hardness	Too high	20.97%	-0.810	0.000
WWG-P*	Toasted intensity	Too low	25.81%	1.463	0.377
WWG-P	Toasted intensity	Too high	9.68%	-0.475	0.000
WWG-P NA	Thickness	Too low	0.00%		
WWG-P	Thickness	Too high	32.26%	-1.190	0.000
WWG-F	Roughness	Too low	17.74%	1.750	0.310
WWG-F	Roughness	Too high	37.10%	-1.380	0.000
WWG-F*	Hardness	Too low	29.03%	1.611	0.468
WWG-F	Hardness	Too high	41.94%	-1.000	0.000
WWG-F*	Toasted intensity	Too low	48.39%	1.447	0.700
WWG-F	Toasted intensity	Too high	11.29%	-1.520	0.000
WWG-F	Thickness	Too low	8.06%	1.900	0.153
WWG-F	Thickness	Too high	37.10%	-1.630	0.000
DWG-P	Roughness	Too low	17.74%	-1.829	0.000
DWG-P	Roughness	Too high	16.13%	2.371	0.382
DWG-P	Hardness	Too low	11.29%	-1.500	0.000
DWG-P*	Hardness	Too high	24.19%	2.300	0.556
DWG-P	Toasted intensity	Too low	30.65%	-1.803	0.000
DWG-P	Toasted intensity	Too high	12.90%	2.079	0.268
DWG-P	Thickness	Too low	6.45%	-1.429	0.000
DWG-P*	Thickness	Too high	25.81%	2.134	0.551
DWG-F	Roughness	Too low	6.45%	-1.244	0.000
DWG-F*	Roughness	Too high	27.42%	2.933	0.804
DWG-F	Hardness	Too low	6.45%	-1.468	0.000
DWG-F	Hardness	Too high	17.74%	3.350	0.594
DWG-F	Toasted intensity	Too low	12.90%	-1.421	0.000
DWG-F*	Toasted intensity	Too high	25.81%	2.829	0.730
DWG-F	Thickness	Too low	1.61%	-1.535	0.000
DWG-F*	Thickness	Too high	29.03%	1.576	0.458
DWB-P	Roughness	Too low	17.74%	-1.026	0.000
DWB-P	Roughness	Too high	9.68%	1.656	0.160
DWB-P	Hardness	Too low	32.26%	-1.295	0.000
DWB-P	Hardness	Too high	1.61%	2.805	0.045
DWB-P	Toasted intensity	Too low	40.32%	-1.394	0.000

DWB-P	Toasted intensity	Too high	3.23%	2.186	0.071
DWB-P	Thickness	Too low	33.87%	-1.143	0.000
DWB-P	Thickness	Too high	11.29%	1.857	0.210
DWB-F	Roughness	Too low	14.52%	-1.111	0.000
DWB-F	Roughness	Too high	35.48%	-2.364	0.000
DWB-F	Hardness	Too low	17.74%	-1.631	0.000
DWB-F*	Hardness	Too high	24.19%	2.344	0.567
DWB-F	Toasted intensity	Too low	6.45%	-0.911	0.000
DWB-F*	Toasted intensity	Too high	43.55%	2.061	0.898
DWB-F	Thickness	Too low	27.42%	-1.459	0.000
DWB-F*	Thickness	Too high	22.58%	2.343	0.529
WM-P	Roughness	Too low	16.13%	-1.344	0.000
WM-P	Roughness	Too high	11.29%	2.927	0.330
WM-P	Hardness	Too low	17.74%	-1.534	0.000
WM-P	Hardness	Too high	3.23%	4.102	0.132
WM-P NA	Toasted intensity	Too low	0.00%		
WM-P	Toasted intensity	Too high	24.19%	0.055	0.013
WM-P	Thickness	Too low	8.06%	-1.320	0.000
WM-P	Thickness	Too high	11.29%	3.051	0.345
WM-F	Roughness	Too low	8.06%	-0.314	0.000
WM-F*	Roughness	Too high	35.48%	2.758	0.979
WM-F	Hardness	Too low	3.23%	1.333	0.043
WM-F*	Hardness	Too high	24.19%	3.133	0.758
WM-F	Toasted intensity	Too low	11.29%	-1.070	0.000
WM-F*	Toasted intensity	Too high	22.58%	2.930	0.662
WM-F	Thickness	Too low	11.29%	-0.932	0.000
WM-F*	Thickness	Too high	27.42%	2.858	0.784

Notes

TPS was calculated separately for each non-JAR direction as p(non-JAR) × positive mean drop, where p(non-JAR) is the proportion of consumers selecting the corresponding "too low" or "too high" category. Mean drop = mean overall liking of consumers rating the attribute as JAR − mean overall liking of consumers in the corresponding non-JAR category. When the mean drop was zero or negative, TPS was set to zero. "Too low" and "too high" deviations were treated separately and were not summed. A penalty was considered relevant for interpretation when the corresponding non-JAR category included at least 20% of consumers and the mean drop exceeded 1 point on the 9-point liking scale. Mean drop was unavailable (NA) only when no consumer selected the corresponding non-JAR category. Asterisks indicate non-JAR deviations considered relevant for interpretation, defined as those selected by ≥20% of consumers and associated with a mean drop in overall liking >1 point on the 9-point hedonic scale."
